# Development, validation, and evaluation of a risk assessment tool for personalized screening of gastric cancer in Chinese populations

**DOI:** 10.1186/s12916-023-02864-0

**Published:** 2023-04-27

**Authors:** Xia Zhu, Jun Lv, Meng Zhu, Caiwang Yan, Bin Deng, Canqing Yu, Yu Guo, Jing Ni, Qiang She, Tianpei Wang, Jiayu Wang, Yue Jiang, Jiaping Chen, Dong Hang, Ci Song, Xuefeng Gao, Jian Wu, Juncheng Dai, Hongxia Ma, Ling Yang, Yiping Chen, Mingyang Song, Qingyi Wei, Zhengming Chen, Zhibin Hu, Hongbing Shen, Yanbing Ding, Liming Li, Guangfu Jin

**Affiliations:** 1 Department of Epidemiology, Center for Global Health, School of Public Health, Nanjing Medical University, 101 Longmian Rd, Nanjing, 211166 China; 2 Jiangsu Key Lab of Cancer Biomarkers, Prevention and Treatment, Collaborative Innovation Center for Cancer Medicine, China International Cooperation Center for Environment and Human Health, Nanjing Medical University, Nanjing, China; 3 Department of Epidemiology and Biostatistics, School of Public Health, Peking University Health Science Center, 38 Xueyuan Road, Beijing, 100191 China; 4 Peking University Center for Public Health and Epidemic Preparedness & Response, Beijing, China; 5 Department of Gastroenterology, The Affiliated Hospital of Yangzhou University, Yangzhou University, 88 Daxue South Rd, Yangzhou, 225000 China; 6 Fuwai Hospital Chinese Academy of Medical Sciences, Beijing, China; 7 Public Health Institute of Gusu School, The Affiliated Suzhou Hospital of Nanjing Medical University, Suzhou, China; 8 Clinical Trial Service Unit and Epidemiological Studies Unit, Nuffield Department of Population Health, University of Oxford, Oxford, UK; 9 Departments of Epidemiology and Nutrition, Harvard University T.H. Chan School of Public Health, Boston, MA USA; 10 Duke Cancer Institute, Duke University Medical Center, Durham, NC USA; 11 Department of Population Health Sciences, Duke University School of Medicine, Durham, NC USA; 12 Jiangsu Key Laboratory of Molecular and Translational Cancer Research, Jiangsu Cancer Hospital, Jiangsu Institute of Cancer Research, The Affiliated Cancer Hospital of Nanjing Medical University, Nanjing, China

**Keywords:** Gastric cancer, Risk prediction, Risk stratification, Screening, Personalized prevention

## Abstract

**Background:**

Effective risk prediction models are lacking for personalized endoscopic screening of gastric cancer (GC). We aimed to develop, validate, and evaluate a questionnaire-based GC risk assessment tool for risk prediction and stratification in the Chinese population.

**Methods:**

In this three-stage multicenter study, we first selected eligible variables by Cox regression models and constructed a GC risk score (GCRS) based on regression coefficients in 416,343 subjects (aged 40–75 years) from the China Kadoorie Biobank (CKB, development cohort). In the same age range, we validated the GCRS effectiveness in 13,982 subjects from another independent Changzhou cohort (validation cohort) as well as in 5348 subjects from an endoscopy screening program in Yangzhou. Finally, we categorized participants into low (bottom 20%), intermediate (20–80%), and high risk (top 20%) groups by the GCRS distribution in the development cohort.

**Results:**

The GCRS using 11 questionnaire-based variables demonstrated a Harrell’s C-index of 0.754 (95% CI, 0.745–0.762) and 0.736 (95% CI, 0.710–0.761) in the two cohorts, respectively. In the validation cohort, the 10-year risk was 0.34%, 1.05%, and 4.32% for individuals with a low (≤ 13.6), intermediate (13.7~30.6), and high (≥ 30.7) GCRS, respectively. In the endoscopic screening program, the detection rate of GC varied from 0.00% in low-GCRS individuals, 0.27% with intermediate GCRS, to 2.59% with high GCRS. A proportion of 81.6% of all GC cases was identified from the high-GCRS group, which represented 28.9% of all the screened participants.

**Conclusions:**

The GCRS can be an effective risk assessment tool for tailored endoscopic screening of GC in China. Risk Evaluation for Stomach Cancer by Yourself (RESCUE), an online tool was developed to aid the use of GCRS.

**Supplementary Information:**

The online version contains supplementary material available at 10.1186/s12916-023-02864-0.

## Background

Gastric cancer (GC) is the fifth most frequently diagnosed cancer and the fourth leading cause of cancer death worldwide [1]. Nearly three-quarters of all new cases and deaths from GC occur in Asian countries, including China, Japan, and Korea [2]. However, among these three countries, the incidence rates of GC are higher in Japan and Korea, whereas the mortality rate is higher in China [2]. This disparity is mainly due to the differences in the early detection of GC, leading to high 5-year survival rates in Japan (60.3%) and Korea (68.9%) but a much lower rate in China (35.9%) [3]. Therefore, screening is critical to improve early detection and treatment and to ultimately reduce GC mortality in China.

Endoscopic screening has been shown to reduce GC mortality by 40% in Asian countries [4]. In Japan, a national GC screening was implemented in 1983, and endoscopic screening was recommended for individuals aged 50 years and older [5]. In Korea, a nationwide screening program was launched in 1999 to screen individuals aged 40 years and older for GC by either upper endoscopy or upper gastrointestinal series examinations [6]. However, in China, there is still no national screening policy or program, because screening in a huge population is cost-prohibitive and requires the capabilities of local doctors and access to available technology. Recently, an endoscopic screening program showed significant reductions in both incidence and mortality of upper gastrointestinal cancer among local permanent residents aged between 40 and 69 years from six high-risk areas of China [7]. Thus, tailored endoscopic screening in high-risk populations represents a more feasible and cost-effective approach in China.

Currently, the consensus on the GC screening in China is to target the subpopulation aged 40 years or older [8]. However, more than 300 million people in China meet the criteria of the consensus, making it impracticable at present [9]. Several prescreening tools prior to a gastroscopy have been developed for GC, which usually combine *Helicobacter pylori* (*H. pylori*) serology tests, serum pepsinogen (PG) I and PG II, and gastrin-17 (G-17) levels [9–11]. Although these tools are effective in identifying high-risk individuals for GC, these serum biomarkers need to be measured in hospitals or other professional institutions and have inconsistent performance in different populations, leading to additional costs and increased difficulty in screening settings.

A number of risk prediction models based on traditional risk factors have been developed for breast cancer [12], colorectal cancer [13], and lung cancer [14]. However, to date, very few risk prediction models have been developed for GC [9, 11, 15, 16], and none has been used for organized screening programs largely due to the lack of external validations required before translation into practice. Herein, leveraging a nationwide prospective cohort, the China Kadoorie Biobank (CKB), we developed a GC risk score (GCRS) based on examination-free variables from questionnaires. We further validated its effectiveness and usefulness in an independent prospective cohort and a real-world cross-sectional endoscopy screening program, respectively. Finally, based on the GCRS, we developed an online tool, named Risk Evaluation for Stomach Cancer by Yourself (RESCUE) [17], to be utilized by the public for GC risk assessment.

## Methods

### Study design and subjects

A three-stage study design was used in the present study (Additional file 1: Fig. S1). In the first stage, the CKB, the largest prospective cohort in China, was used to develop the GCRS. Details of the CKB have been described previously [18, 19]. Briefly, a total of 512,714 participants (aged 30–79 years) were recruited from 10 (5 urban and 5 rural) areas between June 2004 and July 2008. In the present study, we excluded those with GC diagnosed at baseline (*n* = 264), outside the target age range of 40–75 years old (*n* = 81,047), or with missing covariates (*n* = 15,060) and finally included 416,343 eligible subjects in the construction of the GCRS.

In the second stage, the GCRS was validated in an independent prospective cohort from Changzhou of Jiangsu province, China. A total of 20,803 permanent residents aged 35 years or older were enrolled between April 2004 and August 2005 [20]. In this cohort, a total of 13,982 eligible participants remained after excluding those diagnosed with GC at baseline (*n* = 42), outside the age range of 40–75 years old (*n* = 6520), with missing covariates (*n* = 214), or loss to follow-up (*n* = 45).

In the third stage, the GCRS was evaluated in an ongoing upper gastrointestinal disease screening program from Yangzhou of Jiangsu province, China. Permanent residents aged between 40 and 75 years old from eight administrative communities were invited to participate in the program since December 2017. Until March 2022, a total of 5718 participants were recruited. After a face-to-face questionnaire interview and physical examinations, each participant also underwent upper gastrointestinal endoscopy and pathological biopsy. Besides the aforementioned exclusion criteria (*n* = 117), those who lacked pathological biopsy reports (*n* = 175) or had missing covariates (*n* = 78) were also excluded, leaving a total of 5348 participants for the final analysis.

All participants signed a written informed consent on enrollment. Further information on the study details can be found in the Additional file 1: Appendix 1.0 [18–20].

### Procedures

Self-reported information on demographic characteristics, lifestyle, dietary pattern, and medical history was obtained through similar questionnaires in the CKB cohort, the Changzhou cohort, and the Yangzhou screening program. In preliminary analyses of the CKB cohort, the predefined candidate predictors for model derivation were included according to the following criteria: (1) established or probable risk factors of gastric cancer through systematic literature review, (2) established in reported gastric cancer risk prediction models, and (3) available in questionnaires of the CKB. As a result, age [9, 15, 16]; sex [15, 16]; education [21]; smoking [15, 22]; alcohol drinking [22]; consumption of fresh fruits and vegetables [23]; salty food intake [9]; physical activity [22], body mass index (BMI) [22]; medical history of physician-diagnosed cancer [24, 25], gastrointestinal diseases (e.g., peptic ulcer) [26, 27], or diabetes [28]; and family history of cancer in first-degree relatives [22] were identified as candidate predictors.

The primary outcome of the CKB and Changzhou cohort analysis was incident GC as classified by the 10th Revision of the International Classification of Diseases (ICD-10 codes C16). The complete follow-up for the CKB was updated on December 31, 2016. For the Changzhou cohort, three follow-up investigations were performed in 2008–2009, 2012–2013, and 2018–2019, separately. In the Yangzhou screening program, the primary outcome was histopathologically diagnosed GC, and the secondary outcomes included dysplasia (DYS), intestinal metaplasia (IM), atrophic gastritis (AG), and chronic superficial gastritis (SG). All the diagnoses were based on the gastric epithelial neoplasia classification system from the Japanese Research Society for Gastric Cancer (JRSGC) [29]. Detailed information about the definition of risk predictors and outcome assessment in the three studies is detailed in the Additional file 1: Appendix 2.0 and 3.0 [23, 29–34]. Deidentified datasets of the Changzhou cohort and Yangzhou screening program analyzed during the current study are available in Additional file 2.

### Statistical analyses

All participants were assessed for their GC risk since enrollment until the time of GC diagnosis, death, loss to follow-up, or the end of follow-up, whichever occurred first. Cox proportional hazards regression model was used to assess the association between each variable and incident GC risk and to estimate hazard ratios (HRs) with 95% confidence intervals (CIs) in the CKB cohort. Univariate analyses were performed to select potentially effective predictors firstly, and those with *P* < 0.20 were kept for building a multivariate Cox regression model, followed by backward stepwise regression analyses. Based on the final Cox regression model in the CKB cohort, a regression coefficient-based scoring method was adopted to calculate the GCRS. One point was assigned to the predictor with the minimum regression coefficient in the model, and other predictors were assigned with the ratios of corresponding coefficients against the minimum coefficient. The points of predictors were kept to one decimal place and then summed up to generate a GCRS for each participant.

The predicted risk was estimated by using the “predict” function with the type of “expected” from the “survival” package with GCRS as a predictor. The observed GC risk was calculated by the Kaplan-Meier method. Model calibration was assessed by plotting the mean of the predicted probability against the mean of the observed probability of GC at 10 years by the tenth of predicted risk. *R*^2^ was calculated from the linear regression and used to assess the quantitative calibration [35]. Model discrimination was assessed with Harrell’s concordance C (Harrell’s C-index). Receiver operating characteristic (ROC) curves were plotted with all possible GCRSs as cutoff points for the prediction of developing GC within 10 years of follow-up [36]. We also evaluated the model performance separately for 10 study regions. Internal validation of model discrimination was assessed by using the tenfold cross-validation [37, 38].

The absolute risk of GC was projected at three time points (3, 5, and 10 years) by the deciles of the GCRS. Participants were further categorized into low (bottom 20%), intermediate (20–80%), and high (top 20%) risk groups based on the distribution of the GCRS in the CKB cohort, and the corresponding 3-, 5-, and 10-year cumulative incidences were estimated. In the Changzhou cohort and Yangzhou screening program, we calculated the GCRS for each participant blinded to the outcome with the same method used in the CKB cohort. We also estimated the performance of the GCRS corresponding to the deciles as cutoffs in the Yangzhou screening program. Sensitivity, specificity, positive predictive value (PPV), negative predictive value (NPV), and numbers needed to be screened (NNS, one divided by the PPV) were evaluated.

We conducted sensitivity analyses to assess the robustness of our results. Firstly, a simplified model was created based on a subset of strong predictors (the assigned points ≥ 4.0). Secondly, a healthy lifestyle index was generated by integrating five modifiable lifestyle factors (generally weak predictors being assigned points < 4.0), i.e., BMI, smoking, alcohol use, consumption of fresh vegetables and fruits, and salty food intake. Thirdly, we excluded participants who had GC diagnosis within the first year after recruitment to avoid detection bias. Fourthly, in order to avoid the potential interaction between different cancers, we excluded all cancer participants at baseline. Finally, a competing risk model by considering death as a competing event was conducted, since those participants might develop GC thereafter. Additionally, the above sensitivity analyses were conducted by reconstructing GCRS accordingly, and the discrimination and calibration abilities were investigated as well. All *P*-values were two-sided, and *P* < 0.05 was considered statistically significant unless specified otherwise. All statistical analyses were performed by using R version 3.6.3 (R Core Team, Vienna, Austria).

## Results

### Study populations

During a median follow-up of 10.1 years (interquartile range [IQR] 9.2–11.1 years; total 4,107,740 person-years), we documented 3089 incident GC cases in the CKB cohort, while among 13,982 eligible participants in the Changzhou cohort, 329 incident GC cases were diagnosed during a median follow-up of 13.6 years (IQR 13.5–14.4 years; total 182,628 person-years). A total of 49 (0.9%) GC, 163 (3.0%) DYS, 868 (16.2%) IM, and 1626 (30.4%) AG were histologically confirmed in the Yangzhou screening program. The characteristics of the study participants are summarized in Table 1.

**Table 1:** Baseline characteristics and gastric cancer cases in the three studies

Variables	CKB development cohort			Changzhou validation cohort			Yangzhou screening program		
	Total (*n* = 416,343), no. (%)	Cases (*n* = 3089), no.	Incidence rate (per 100,000 person-years)	Total (*n* = 13,982), no. (%)	Cases (*n* = 329), no.	Incidence rate (per 100,000 person-years)	Total (*n* = 5348), no. (%)	Cases (*n* = 49), no.	Detection rate, %
Age at baseline, mean (SD), years	54.29 (9.14)			54.27 (8.97)			57.54 (8.19)		
40–44	83,099 (19.96)	168	19.88	2509 (17.94)	19	55.43	280 (5.24)	0	0.00
45–49	67,379 (16.18)	236	34.31	2484 (17.77)	31	92.27	708 (13.24)	1	0.14
50–54	85,415 (20.52)	473	55.00	2827 (20.22)	54	143.38	1178 (22.03)	4	0.34
55–59	67,407 (16.19)	593	89.01	2518 (18.01)	68	207.45	881 (16.47)	2	0.23
60–64	47,091 (11.31)	572	125.86	1612 (11.53)	70	341.75	929 (17.37)	13	1.40
65–69	38,720 (9.30)	570	159.27	1222 (8.74)	58	394.80	1031 (19.28)	21	2.04
70–75	27,232 (6.54)	477	201.92	810 (5.79)	29	317.33	341 (6.38)	8	2.35
Sex									
Women	244,810 (58.80)	1033	42.20	8010 (57.29)	95	89.38	3132 (58.56)	7	0.22
Men	171,533 (41.20)	2056	123.87	5972 (42.71)	234	306.53	2216 (41.44)	42	1.90
Education									
College or above	21,145 (5.08)	102	48.71	64 (0.46)	1	119.27	122 (2.28)	0	0.00
High school	63,402 (15.23)	310	49.18	1269 (9.08)	17	99.52	475 (8.88)	3	0.63
Middle school	109,701 (26.35)	664	61.01	5432 (38.85)	108	150.00	1628 (30.44)	12	0.74
Illiterate or primary school	222,095 (53.34)	2013	92.35	7217 (51.62)	203	218.97	3123 (58.40)	34	1.09
BMI, mean (SD)	23.76 (3.39)			23.54 (3.35)			24.43 (3.04)		
≥ 18.5	398,633 (95.75)	2885	73.16	13,299 (95.12)	312	179.12	5258 (98.32)	46	0.87
< 18.5	17,710 (4.25)	204	124.26	683 (4.88)	17	201.37	90 (1.68)	3	3.33
Pack-years of smoking									
Never (0 pack-year)	278,156 (66.81)	1389	50.09	9544 (68.26)	160	127.29	3728 (69.71)	18	0.48
> 0 to < 20 pack-years	55,078 (13.23)	559	104.18	1406 (10.06)	39	212.39	679 (12.70)	15	2.21
≥ 20 pack-years	83,109 (19.96)	1141	142.94	3032 (21.69)	130	337.07	941 (17.60)	16	1.70
Alcohol drinking per day^a^									
Never or light	379,928 (91.25)	2598	69.27	11,218 (80.23)	224	152.40	4716 (88.18)	39	0.83
Moderate or heavy	36,415 (8.75)	491	137.46	2764 (19.77)	105	294.57	632 (11.82)	10	1.58
Intake of fresh vegetables and fruits^b^									
Frequent	113,736 (27.32)	695	61.29	4589 (32.82)	89	146.21	1586 (29.66)	9	0.57
Occasional	302,607 (72.68)	2394	80.51	9393 (67.18)	240	197.12	3762 (70.34)	40	1.06
Intake of salty foods^c^									
Occasional	320,109 (76.89)	2105	66.90	7494 (53.60)	177	181.19	4646 (86.87)	41	0.88
Frequent	96,234 (23.11)	984	102.37	6488 (46.40)	152	178.95	702 (13.13)	8	1.14
Previous cancer diagnosis									
No	414,227 (99.49)	3042	74.39	13,854 (99.08)	324	178.84	5312 (99.33)	49	0.92
Yes	2116 (0.51)	47	256.70	128 (0.92)	5	341.60	36 (0.67)	0	0.00
Family history of cancer in first-degree relatives									
No	341,680 (82.07)	2328	69.16	11,121 (79.54)	247	169.73	3556 (66.49)	29	0.82
Yes	74,663 (17.93)	761	102.60	2861 (20.46)	82	220.99	1792 (33.51)	20	1.12
History of peptic ulcer									
No	399,248 (95.89)	2844	72.19	13,822 (98.86)	321	177.71	4886 (91.36)	44	0.90
Yes	17,095 (4.11)	245	145.81	160 (1.14)	8	400.69	462 (8.64)	5	1.08

*SD* Standard deviation, *BMI* Body mass index, *CKB* China Kadoorie Biobank

^a^Never or light alcohol drinking was defined as alcohol intake less than 25 g/day in men and 15 g/day in women in the past year, otherwise was moderate or heavy alcohol drinking

^b^In the CKB cohort, frequent intake of fresh vegetables and fruits was defined as eating vegetables every day and fruits ≥ 4 days per week or eating fruits every day and vegetables ≥ 4 days per week, otherwise was occasional. In the Changzhou cohort and Yangzhou screening program, frequent intake was defined as eating vegetables every day and fruits at least 3 days per week or eating fruits every day and vegetables at least 3 days per week, otherwise was occasional

^c^In the CKB cohort, frequent intake of salty foods was defined as eating preserved salty vegetables ≥ 4 days per week, otherwise was occasional. In the Changzhou and Yangzhou studies, frequent intake of salty foods was defined as eating preserved salty vegetables at least 3 days per week

### Development of the GCRS in the CKB cohort

In the CKB cohort, after the stepwise regression analysis, 11 of 13 variables were identified to be significantly (*P* < 0.05) and independently associated with the risk of GC (Table 2 and Additional file 1: Table S1). Based on the multivariate Cox regression model, one point was assigned to the variable of consumption of fresh vegetables and fruits that showed the minimum coefficient, and risk points were then assigned to other included variables for the GCRS calculation accordingly (Table 2). Similar estimates were yielded in sensitivity analyses when excluding weak variables in the simplified model or integrating lifestyle factors as an index (Additional file 1: Tables S2 and S3). Besides, the estimated HRs and assigned points were largely unchanged when excluding participants who had GC diagnosis within the first year after recruitment, excluding participants who had cancer at baseline or performing competing risk model (Additional file 1: Tables S4-S6).

**Table 2:** Detailed descriptions of the model predictors in the CKB cohort and corresponding risk points

Variables	Cases/person-years	Regression coefficient	HR (95% CI)	*P*-value	Points assigned
Age at baseline, years					
40–44	168/845,021		Reference		0.0
45–49	236/687,811	0.47	1.59 (1.31 to 1.94)	< 0.001	4.6
50–54	473/860,063	0.87	2.39 (2.00 to 2.86)	< 0.001	8.6
55–59	593/666,252	1.33	3.79 (3.18 to 4.52)	< 0.001	13.2
60–64	572/454,473	1.67	5.29 (4.43 to 6.30)	< 0.001	16.5
65–69	570/357,890	1.90	6.71 (5.62 to 8.01)	< 0.001	18.8
70–75	477/236,230	2.13	8.42 (7.02 to 10.11)	< 0.001	21.1
Sex					
Women	1033/2,447,930		Reference		0.0
Men	2056/1,659,810	0.87	2.39 (2.15 to 2.66)	< 0.001	8.6
Education					
College or above	102/209,414		Reference		0.0
High school	310/630,316	0.40	1.49 (1.19 to 1.87)	< 0.001	4.0
Middle school	664/1,088,309	0.54	1.72 (1.39 to 2.12)	< 0.001	5.3
Illiterate or primary school	2013/2,179,701	0.67	1.95 (1.58 to 2.39)	< 0.001	6.6
BMI					
≥ 18.5	2885/3,943,563		Reference		0.0
< 18.5	204/164,177	0.25	1.28 (1.11 to 1.48)	< 0.001	2.5
Pack-years of smoking					
Never (0 pack-year)	1389/2,772,929		Reference		0.0
> 0 to < 20 pack-years	559/536,569	0.12	1.13 (1.00 to 1.27)	0.049	1.2
≥ 20 pack-years	1141/798,243	0.22	1.25 (1.12 to 1.39)	< 0.001	2.2
Alcohol drinking per day^a^					
Never or light	2598/3,750,542		Reference		0.0
Moderate or heavy	491/357,198	0.17	1.19 (1.07 to 1.32)	0.001	1.7
Intake of fresh vegetables and fruits^b^					
Frequent	695/1,134,030		Reference		0.0
Occasional	2394/2,973,710	0.10	1.11 (1.01 to 1.21)	0.027	1.0
Intake of salty foods^c^					
Occasional	2105/3,146,493		Reference		0.0
Frequent	984/961,247	0.40	1.50 (1.39 to 1.62)	< 0.001	4.0
Previous cancer diagnosis					
No	3042/4,089,431		Reference		0.0
Yes	47/18,309	1.09	2.96 (2.22 to 3.95)	< 0.001	10.7
Family history of cancer in first-degree relatives					
No	2328/3,366,039		Reference		0.0
Yes	761/741,701	0.40	1.50 (1.38 to 1.63)	< 0.001	4.0
History of peptic ulcer					
No	2844/3,939,709		Reference		0.0
Yes	245/168,031	0.47	1.59 (1.40 to 1.82)	< 0.001	4.6

*HR* Hazard ratio, *CI* Confidence interval, *BMI* Body mass index, *CKB* China Kadoorie Biobank

^a^Never or light alcohol drinking was defined as alcohol intake less than 25 g/day in men and 15 g/day in women in the past year, otherwise was moderate or heavy alcohol drinking

^b^Frequent intake of fresh vegetables and fruits was defined as eating vegetables every day and fruits ≥ 4 days per week or eating fruits every day and vegetables ≥ 4 days per week, otherwise was occasional

^c^Frequent intake of salty foods was defined as eating preserved salty vegetables ≥ 4 days per week, otherwise was occasional

**Fig. 1:**
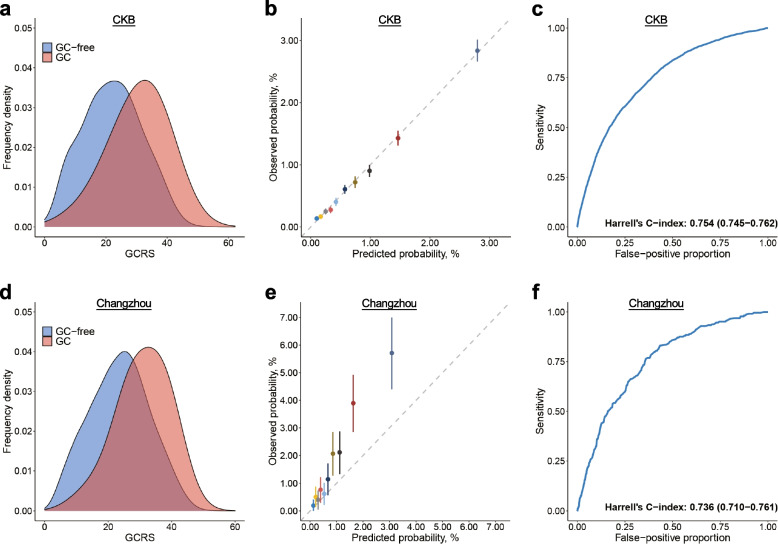
Distribution, calibration, and discrimination of the GCRS in the CKB and Changzhou cohorts. **a**, **d** Distribution of the GCRS between incident gastric cancer (GC) cases and GC-free participants in the **a** CKB and **d** Changzhou cohorts. **b**, **e** The observed 10-year probability of GC with 95% CIs was estimated by the Kaplan-Meier method within deciles of GCRS-based model-predicted probability in the **b** CKB and **e** Changzhou cohorts. **c**, **f** Receiver operating characteristic curve at 10 years in the **c** CKB cohort (Harrell’s C-index of 0.754, 95% CI 0.745–0.762) and **f** Changzhou cohort (Harrell’s C-index of 0.736, 95% CI 0.710–0.761). GCRS, gastric cancer risk score; CKB, China Kadoorie Biobank

A significantly higher GCRS was observed for those with incident GC (30.6 ± 8.9) compared with those GC-free (22.1 ± 9.6) (Fig. 1a). The incidence of GC increased significantly with GCRS (*P*_trend_ < 0.001) (Additional file 1: Table S7 and Fig. S2). The GCRS by deciles was calibrated well with the observed 10-year GC risk, with an *R*^2^ coefficient of 0.998, indicating a good calibration for the GCRS (Fig. 1b). The ROC curve of the GCRS indicated relatively high discrimination for the 10-year risk of incident GC, with Harrell’s C-index of 0.754 (95% CI, 0.745–0.762) (Fig. 1c). There were slight differences in the discrimination performances of the model across different study regions (Additional file 1: Table S8). Internal tenfold cross-validation showed a similar Harrell’s C-index (Additional file 1: Table S9).

### Validation of the GCRS in the Changzhou cohort

In the Changzhou cohort, we also observed a higher distribution of the GCRS in incident GC cases (30.9 ± 8.2) compared with those GC-free (23.6 ± 9.3) (Fig. 1d). The GCRS was significantly associated with an increased incidence of GC (Additional file 1: Table S10 and Fig. S3). The GCRS agreed well with the observed risk of incident GC with an *R*^2^ coefficient of 0.965 (Fig. 1e), which also showed a fairly good discrimination capability (Harrell’s C-index: 0.736, 95% CI, 0.710–0.761) (Fig. 1f). However, the incidence rate of GC is much higher in the Changzhou cohort than in the CKB (180/100,000 person-years vs 75/100,000 person-years); therefore, the predicted probability was much lower than observed (Fig. 1e). The performance of the GCRS did not change substantially in the sensitivity analyses (Additional file 1: Figs. S4-S8).

### GCRS categories and absolute risk of incident GC

**Fig. 2:**
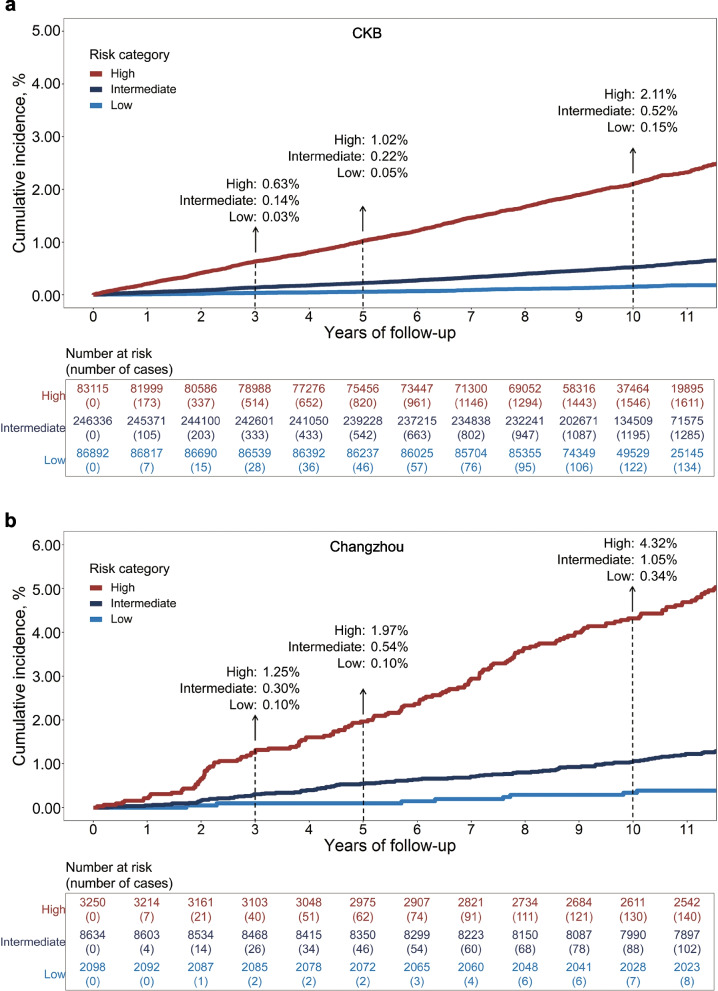
Inverted Kaplan-Meier plot of incident GC in the CKB and Changzhou cohorts by GCRS. Participants in the **a** CKB and **b** Changzhou cohorts were divided into low (bottom 20% of the GCRS: ≤ 13.6), intermediate (20–80%: 13.7~30.6), and high (top 20%: ≥ 30.7) risk groups. The cumulative incidence of GC was calculated by using the Kaplan-Meier method. The risk table under the plot showed the number at risk and the corresponding cumulative number of incident GC cases at years of follow-up. GCRS, gastric cancer risk score; CKB, China Kadoorie Biobank

In the CKB cohort, by comparing participants at the top decile to the bottom decile of the GCRS, we found that the HRs were 33.90 (95% CI, 18.61–61.77), 34.02 (95% CI, 21.00–55.13) and 20.26 (95% CI, 15.33–26.78) at 3, 5, and 10 years, respectively (Additional file 1: Table S11). We further divided participants into low (bottom 20% of the GCRS: ≤ 13.6), indeterminate (20–80%: 13.7~30.6), and high (top 20%: ≥ 30.7) GCRS groups in the CKB cohort and found that their 10-year incidence of GC was 0.15%, 0.52%, and 2.11% (Fig. 2a), respectively. By using the same cutoffs, we found that participants in the Changzhou cohort also showed a differentiated risk of incident GC across the three risk levels (Fig. 2b), with a 10-year incidence of 0.34%, 1.05%, and 4.32%, respectively. Individuals in the high risk group accounted for 53.2% and 52.0% of all GC cases in the CKB and Changzhou cohorts, respectively (Additional file 1: Tables S7 and S10).

### Application of the GCRS in the Yangzhou endoscopy screening program

**Fig. 3:**
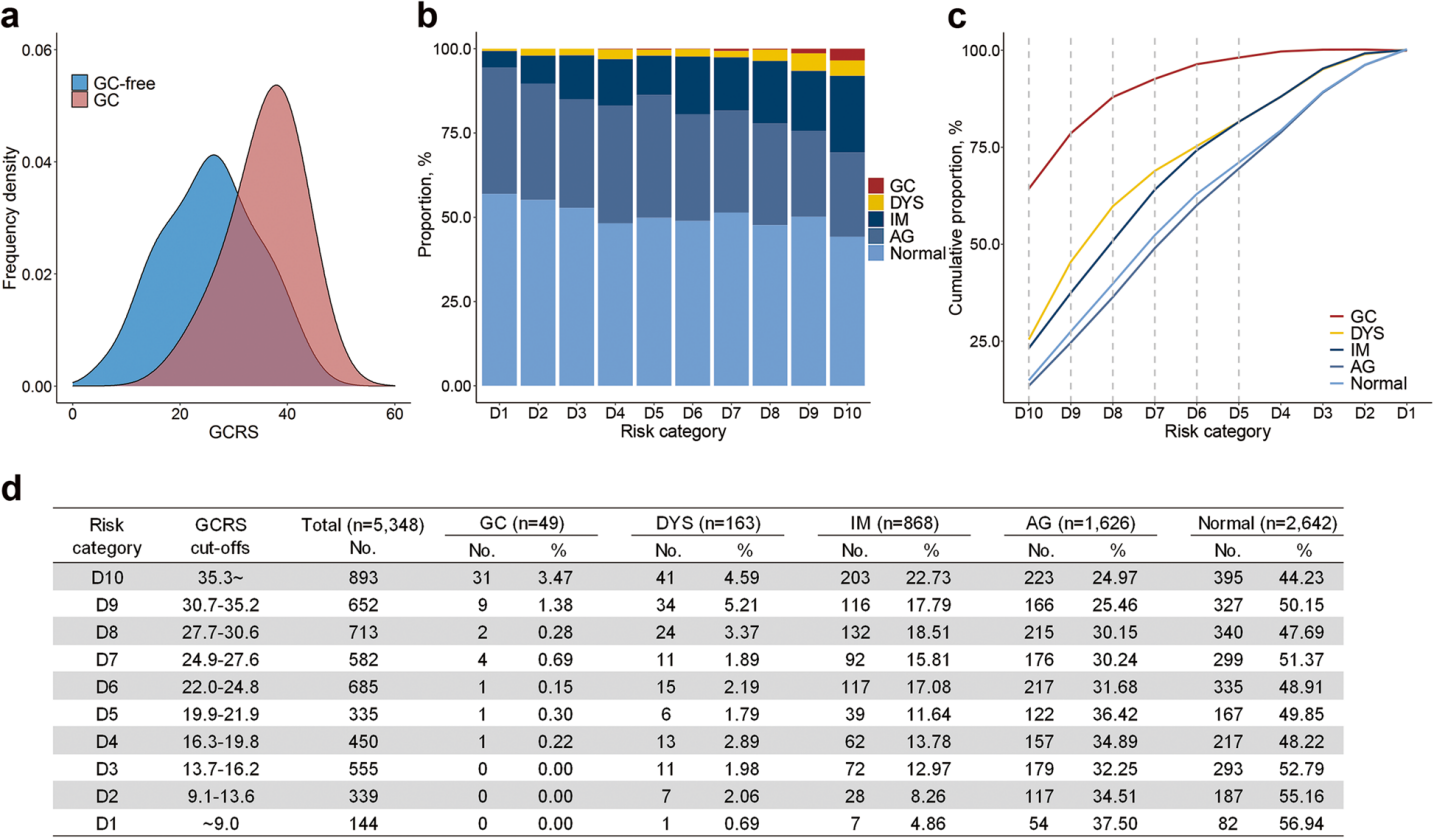
Comparison of pathological biopsy reports by GCRS in the Yangzhou screening program. **a** Distributions of the GCRS between participants who were diagnosed with gastric cancer (GC) and those who were GC-free. **b** Proportion of different lesions in each risk category. Ten risk categories (D1~D10) were based on the same cutoffs of the GCRS deciles from the CKB. **c** Cumulative proportion was calculated by dividing the number of each lesion accumulated to this category by the total number of this lesion. Ten risk categories were based on the same cutoffs of the GCRS deciles from the CKB. **d** Risk table of different lesions in the Yangzhou screening program. GCRS, gastric cancer risk score; DYS, dysplasia; IM, intestinal metaplasia; AG, atrophic gastritis. “Normal” biopsy report includes the diagnosis of chronic superficial gastritis or no lesion

Then, we applied the GCRS to the endoscopy screening program in Yangzhou and observed a higher GCRS in newly diagnosed GC cases than that in GC-free participants (35.9 ± 6.3 vs 25.6 ± 9.0) (Fig. 3a). We observed that the overall detection rates were 0.9%, 3.0%, and 16.2% for GC, DYS, and IM, respectively, which all gradually increased as the GCRS increased (Fig. 3b). Among high-risk (GCRS ≥ 30.7) individuals who accounted for 28.9% of all screening participants (1545 of 5348), 81.6% (40 of 49) of all GC cases, 46.0% (75 of 163) of DYS, and 36.8% (319 of 868) of IM were detected (Fig. 3c and Additional file 1: Table S12). Overall, the detection rate of GC was 2.59% (40 of 1545) and 0.27% (9 of 3320) in participants at high (GCRS ≥ 30.7) and intermediate risk (GCRS: 13.7~30.6), respectively, and no GC cases were detected in those at low risk (GCRS ≤ 13.6) (Fig. 3d). The performance of the GCRS across different predicted risk cutoffs in the Yangzhou screening program was shown in Additional file 1: Table S13. In the sensitivity analyses, similar detection rates were observed in the Yangzhou screening program (Additional file 1: Tables S14-S18).

### RESCUE: a web-based GC risk assessment tool

We presented the risk scoring method of the GCRS (Table 2) online as an easily and freely available tool named RESCUE [17] to allow the general population to quantitatively estimate their risk of GC over the next 3, 5, and 10 years (Additional file 1: Table S19). We also provided tailored lifestyle and screening recommendations according to each individual’s risk profile.

## Discussion

In the present study, by using the largest nationwide prospective cohort in China, we developed a GC risk assessment tool of GCRS based on eleven variables that could be easily determined without any physical examinations. We validated the GCRS with good calibration and discrimination in the independent Changzhou cohort, demonstrating the great potential of GCRS for GC risk prediction and stratification. When applying the GCRS to a real-world endoscopy screening program, we detected approximately 80% of all the identified GC cases in about one-quarter of individuals with high GCRS. To the best of our knowledge, the present study is the first to provide a questionnaire-based GC risk assessment tool based on a large-scale cohort study that can be used for risk stratification in an endoscopic screening setting of the Chinese population.

To date, several risk-prediction models have been developed for GC, but few are translated into practice. For example, the Japan Public Health Center-based Prospective Study (JPHC Study) developed a prediction model including age, sex, smoking status, consumption of high-salt food, family history of gastric cancer, *H. pylori* antibody, and serum pepsinogen, which resulted in a C-statistic of 0.768 for discrimination [15]. In China, there are two risk prediction models for GC, predominantly based on serum PG I, PG II, gastrin-17 (G-17), and anti-*H. pylori* antibody, which were developed in a population-based follow-up study [11] and a hospital-based cross-sectional study [9], respectively. These two models also showed good discrimination (C-statistic of 0.803 and area under the curve of 0.76, respectively). However, these risk prediction models, mainly based on one study population, have a potential risk of over-fitting and should be subjected to rigorous external validations in the future. Of note, these abovementioned models based on serology tests not only add additional costs but also increase the degree of screening complexity, which may decrease the overall participation and efficiency. In the present study, we developed the questionnaire-based GCRS by using the largest Chinese cohort and independently validated the tool in an external cohort with good discrimination (Harrell’s C-index: 0.736). The large sample size and rigorous design ensured the quality and applicability of our GC risk assessment tool, which may be useful for tailored screening practices in the general population.

Although screening by endoscopy could reduce the mortality of GC [4, 7], the availability of endoscopic instruments and expertise for mass screening remains questionable and impractical. Even though some countries, such as Japan and Korea, have implemented a national GC screening program [5, 6], most have adopted screening approaches for high-risk populations. The initial prescreening tools, generally based on risk prediction models, provide a tailored screening for the general population. In the present study, we evaluated the initial GCRS in the Yangzhou screening program and found that 81.6% of the identified GC cases were correctly allocated to undergo endoscopy in the at-high-risk individuals who accounted for only about one-quarter of all screenings; moreover, none of the GC cases was detected in participants at low risk, suggesting that the low-risk populations could also be identified reliably. Thus, the developed GCRS may be employed to a tailored endoscopy screening, which could substantially decrease endoscopy workload and cost, compared with endoscopy for all. However, the incidence rate of GC changed remarkably in different areas across China [39], while the developed GCRS may represent the average level of the Chinese populations. Therefore, further external validation with re-calibrated estimates based on local incidence would be necessary for clinical use [40], especially for setting actionable cut points in different areas of China.

Nevertheless, additional studies are warranted to address several concerns regarding the applicability of the GCRS. First, although the GCRS was developed, replicated, and evaluated in twelve geographic areas of China, the tool needs to be evaluated or optimized in other areas or populations. For example, efforts are required to evaluate the generalizability of the GCRS to hospital-based screening. Second, the GCRS may help inform decision-making for GC screening, but several questions remain to be addressed, including optimal cutoff points of risk stratification, starting and stopping ages, and intensity of screening. Third, the prevalence rate of GC in the CKB was lower than expected, which was probably due to volunteer bias that individuals with GC were not inclined to attend the survey in the CKB at baseline. Nevertheless, the prevalent GC cases might be undetected through questionnaires, which could also contribute to the low prevalence rate and lead to inaccurate estimates of predictors. Fourth, although previous cancer diagnosis was used in this study as a predictor for GC, which was in line with that in lung cancer [41], additional studies are warranted to explore the potential benefit of endoscopic screening in prevalent cancer patients. Fifth, concern still exists regarding whether or how much the GCRS-directed screening can improve the cost-effectiveness of endoscopic screening, compared with the current “one-size-fits-all” approach, which needs to be further assessed in future studies. At last, *H. pylori* infection is the most important risk factor of GC, and we have also reported that a polygenic risk score with 112 genetic variants is effective for risk stratification of GC [23]. However, the information was not available in the discovery and validation cohorts in this study. Therefore, additional studies are needed to develop a comprehensive score with the GCRS, *H. pylori* infection status, polygenic risk score, and other serum biomarkers (e.g., PG I, PG II, and gastrin-17) to further optimize the risk prediction of GC. Moreover, the utility of these scores needs to be evaluated in endoscopy screening practices.

Several limitations of the present study should be noted. First, the lifestyle and personal history information was self-reported at baseline, which may cause some misclassifications and have biased the risk estimates of variables included in the GCRS. Second, we only evaluated the overall GC risk, but the risk estimate might differ depending on tumor location, stage, and subtype that were not obtained with details in the follow-up of cohorts. Third, *H. pylori* infection, the most important risk factor of gastric cancer [42], and family history of upper gastrointestinal cancers [43] were unavailable in the development and validation cohorts and thus not included in the GCRS.

## Conclusions

Based on a three-stage design, we reported a high-performance GC risk assessment tool GCRS that can be easily accessible to the general population. This may be useful for participants to be aware of their GC risk and thus to adopt healthy lifestyles to reduce GC risk. Importantly, this tool can be integrated into health management or physical examination systems and be used to direct individuals to a tailored endoscopy screening by risk stratification. The web-based GCRS, i.e., RESCUE, is now available with risk prediction and recommendations for lifestyle changes and a tailored endoscopy screening. These efforts are likely to facilitate personalized GC prevention and lead to reductions in GC incidence and mortality in China.

## Supplementary Information

**Additional file 1: Appendix 1.0.** Study design and subjects. **Appendix 2.0.** Assessment of risk factors. **Appendix 3.0.** Definition of GC cases. **Table S1.** Results of univariate Cox regression analysis in the CKB cohort. **Table S2.** Results of multivariate Cox regression model and corresponding risk points in sensitivity analysis 1: excluding weak variables in the simplified model. **Table S3.** Results of multivariate Cox regression model and corresponding risk points in sensitivity analysis 2: integrating lifestyle factors as an index. **Table S4.** Results of multivariate Cox regression model and corresponding risk points in sensitivity analysis 3: excluding participants who had GC diagnosis within the first year after recruitment. **Table S5.** Results of multivariate Cox regression model and corresponding risk points in sensitivity analysis 4: excluding participants who had cancer at baseline. **Table S6.** Results and corresponding risk points in sensitivity analysis 5: competing risk model by considering death as a competing event. **Table S7.** Risk categories by deciles of the GCRS in the CKB cohort. **Table S8.** Internal validation of the GCRS in different regions of CKB. **Table S9.** Harrell’s C-index of the GCRS from ten-fold cross validation in the CKB cohort. **Table S10.** Risk categories of the GCRS in the Changzhou cohort. **Table S11.** Risk categories and associated 3-year, 5-year, and 10-year risk of incident GC derived from CKB. **Table S12.** Risk categories of different gastric lesions in the Yangzhou screening program. **Table S13.** Performance of the GCRS across different predicted risk cutoffs in the Yangzhou screening program. **Table S14.** Risk categories of different gastric lesions in the Yangzhou screening program in sensitivity analysis 1: excluding weak variables in the simplified model. **Table S15.** Risk categories of different gastric lesions in the Yangzhou screening program in sensitivity analysis 2: integrating lifestyle factors as an index. **Table S16.** Risk categories of different gastric lesions in the Yangzhou screening program in sensitivity analysis 3: excluding participants who had GC diagnosis within the first year after recruitment. **Table S17.** Risk categories of different gastric lesions in the Yangzhou screening program in sensitivity analysis 4: excluding participants who had cancer at baseline. **Table S18.** Risk categories of different gastric lesions in the Yangzhou screening program in sensitivity analysis 5: competing risk model. **Table S19.** The GCRS and corresponding 3-year, 5-year, and 10-year risk of incident GC derived from the CKB cohort. **Fig. S1.** Study design and eligible participants’ selection procedures in three studies. **Fig. S2.** The relationship of the GCRS with incident GC risk in the CKB cohort. **Fig. S3.** The relationship of the GCRS with incident GC risk in the Changzhou cohort. **Fig. S4.** Calibration and discrimination of the GCRS in sensitivity analysis 1: excluding weak variables in the simplified model. **Fig. S5.** Calibration and discrimination of the GCRS in sensitivity analysis 2: integrating lifestyle factors as an index. **Fig. S6.** Calibration and discrimination of the GCRS in sensitivity analysis 3: excluding participants who had GC diagnosis within the first year after recruitment. **Fig. S7.** Calibration and discrimination of the GCRS in sensitivity analysis 4: excluding participants who had cancer at baseline. **Fig. S8.** Calibration and discrimination of the GCRS in sensitivity analysis 5: competing risk model.

**Additional file 2.** Deidentified datasets of the Changzhou cohort and Yangzhou screening program.

## Data Availability

Details of how to access the China Kadoorie Biobank data are available from https://www.ckbiobank.org/data-access. Deidentified individual participant datasets of the Changzhou cohort and Yangzhou screening program analyzed during the current study (including data dictionaries) are freely available in Additional file 2.

## Abbreviations

AG: Atrophic gastritis
BMI: Body mass index
CI: Confidence interval
CKB: China Kadoorie Biobank
DYS: Dysplasia
G-17: Gastrin-17
GC: Gastric cancer
GCRS: Gastric cancer risk score
*H. pylori*: *Helicobacter pylori*
Harrell’s C-index: Harrell’s concordance statistic
HR: Hazard ratio
ICD: International Classification of Diseases
IM: Intestinal metaplasia
IQR: Interquartile range
JPHC Study: Japan Public Health Center-based Prospective Study
JRSGC: Japanese Research Society for Gastric Cancer
NNS: Numbers needed to be screened
NPV: Negative predictive value
PG II: Pepsinogen II
PGI: Pepsinogen I
PPV: Positive predictive value
RESCUE: Risk Evaluation for Stomach Cancer by Yourself
ROC: Receiver operating characteristic
SD: Standard deviation
SG: Chronic superficial gastritis

## References

[1] Sung H, Ferlay J, Siegel RL, Laversanne M, Soerjomataram I, Jemal A (2021). Global Cancer Statistics 2020: GLOBOCAN estimates of incidence and mortality worldwide for 36 cancers in 185 countries. CA Cancer J Clin.

[2] Cancer Today. https://gco.iarc.fr/today/home. Assessed Nov 2021.

[3] Allemani C, Matsuda T, Di Carlo V, Harewood R, Matz M, Nikšić M (2018). Global surveillance of trends in cancer survival 2000–14 (CONCORD-3): analysis of individual records for 37 513 025 patients diagnosed with one of 18 cancers from 322 population-based registries in 71 countries. Lancet.

[4] Zhang X, Li M, Chen S, Hu J, Guo Q, Liu R (2018). Endoscopic screening in Asian countries is associated with reduced gastric cancer mortality: a meta-analysis and systematic review. Gastroenterology.

[5] Hamashima C, Systematic Review Group and Guideline Development Group for Gastric Cancer Screening Guidelines (2018). Update version of the Japanese guidelines for gastric cancer screening. Jpn J Clin Oncol.

[6] Jun JK, Choi KS, Lee H-Y, Suh M, Park B, Song SH (2017). Effectiveness of the Korean National Cancer Screening Program in reducing gastric cancer mortality. Gastroenterology.

[7] Chen R, Liu Y, Song G, Li B, Zhao D, Hua Z (2021). Effectiveness of one-time endoscopic screening programme in prevention of upper gastrointestinal cancer in China: a multicentre population-based cohort study. Gut.

[8] National Clinical Research Center for Digestive Diseases, Chinese Society of Digestive Endoscopology, Chinese Society of Health Management (2018). China Consensus on the Protocol of Early Gastric Cancer Screening (Shanghai, 2017).. Chin J Gastroenterol.

[9] Cai Q, Zhu C, Yuan Y, Feng Q, Feng Y, Hao Y (2019). Development and validation of a prediction rule for estimating gastric cancer risk in the Chinese high-risk population: a nationwide multicentre study. Gut.

[10] Yamaguchi Y, Nagata Y, Hiratsuka R, Kawase Y, Tominaga T, Takeuchi S (2016). Gastric cancer screening by combined assay for serum anti-Helicobacter pylori IgG antibody and serum pepsinogen levels–the ABC method. Digestion.

[11] Tu H, Sun L, Dong X, Gong Y, Xu Q, Jing J (2017). A serological biopsy using five stomach-specific circulating biomarkers for gastric cancer risk assessment: a multi-phase study. Am J Gastroenterol..

[12] Louro J, Posso M, Hilton Boon M, Román M, Domingo L, Castells X (2019). A systematic review and quality assessment of individualised breast cancer risk prediction models. Br J Cancer.

[13] Robertson DJ, Ladabaum U (2019). Opportunities and challenges in moving from current guidelines to personalized colorectal cancer screening. Gastroenterology.

[14] Muller DC, Johansson M, Brennan P (2017). Lung cancer risk prediction model incorporating lung function: development and validation in the UK Biobank prospective cohort study. J Clin Oncol.

[15] Charvat H, Sasazuki S, Inoue M, Iwasaki M, Sawada N, Shimazu T (2016). Prediction of the 10-year probability of gastric cancer occurrence in the Japanese population: the JPHC study cohort II. Int J Cancer.

[16] Iida M, Ikeda F, Hata J, Hirakawa Y, Ohara T, Mukai N (2018). Development and validation of a risk assessment tool for gastric cancer in a general Japanese population. Gastric Cancer.

[17] RESCUE (Risk Evaluation for Stomach Cancer by Yourself). http://ccra.njmu.edu.cn/rescue/web.

[18] Chen Z, Lee L, Chen J, Collins R, Wu F, Guo Y (2005). Cohort profile: the Kadoorie Study of Chronic Disease in China (KSCDC). Int J Epidemiol.

[19] Chen Z, Chen J, Collins R, Guo Y, Peto R, Wu F (2011). China Kadoorie Biobank of 0.5 million people: survey methods, baseline characteristics and long-term follow-up. Int J Epidemiol.

[20] Chen W, Lu F, Liu S-J, Du J-B, Wang J-M, Qian Y (2012). Cancer risk and key components of metabolic syndrome: a population-based prospective cohort study in Chinese. Chin Med J (Engl).

[21] Kawakatsu Y, Koyanagi YN, Oze I, Kasugai Y, Morioka H, Yamaguchi R (2020). Association between socioeconomic status and digestive tract cancers: a case-control study. Cancers (Basel).

[22] Eom BW, Joo J, Kim S, Shin A, Yang H-R, Park J (2015). Prediction model for gastric cancer incidence in Korean population. PloS One.

[23] Jin G, Lv J, Yang M, Wang M, Zhu M, Wang T (2020). Genetic risk, incident gastric cancer, and healthy lifestyle: a meta-analysis of genome-wide association studies and prospective cohort study. Lancet Oncol.

[24] Ji J, Hemminki K (2006). Second gastric cancers among patients with primary sporadic and familial cancers in Sweden. Gut.

[25] Morais S, Antunes L, Bento MJ, Lunet N (2020). Second primary gastric cancers in a region with an overall high risk of gastric cancer. Gac Sanit.

[26] Cao M, Li H, Sun D, Lei L, Ren J, Shi J (2020). Classifying risk level of gastric cancer: evaluation of questionnaire-based prediction model. Chin J Cancer Res.

[27] Hansson LE, Nyrén O, Hsing AW, Bergström R, Josefsson S, Chow WH (1996). The risk of stomach cancer in patients with gastric or duodenal ulcer disease. N Engl J Med.

[28] Sekikawa A, Fukui H, Maruo T, Tsumura T, Okabe Y, Osaki Y (2014). Diabetes mellitus increases the risk of early gastric cancer development. Eur J Cancer.

[29] Japanese Gastric Cancer Association (1998). Japanese classification of gastric carcinoma - 2nd English edition.. Gastric Cancer.

[30] Lv J, Chen W, Sun D, Li S, Millwood IY, Smith M (2015). Gender-specific association between tobacco smoking and central obesity among 0.5 million Chinese people: the China Kadoorie Biobank Study. PloS One.

[31] Millwood IY, Li L, Smith M, Guo Y, Yang L, Bian Z (2013). Alcohol consumption in 0.5 million people from 10 diverse regions of China: prevalence, patterns and socio-demographic and health-related correlates.. Int J Epidemiol.

[32] Huang F, Wang Z, Wang L, Wang H, Zhang J, Du W (2019). Evaluating adherence to recommended diets in adults 1991–2015: revised China Dietary Guidelines Index. Nutr J.

[33] Yang YX, Wang XL, Leong PM, Zhang HM, Yang XG, Kong LZ (2018). New Chinese dietary guidelines: healthy eating patterns and food-based dietary recommendations. Asia Pac J Clin Nutr.

[34] Chinese Society of Digestive Endoscopy (2014). Consensus on screening and endoscopic diagnosis and treatment of early gastric cancer in China (Changsha, 2014). Zhonghua Xiao Hua Nei Jing Za Zhi..

[35] Lo SN, Ma J, Scolyer RA, Haydu LE, Stretch JR, Saw RPM (2020). Improved risk prediction calculator for sentinel node positivity in patients with melanoma: the Melanoma Institute Australia Nomogram. J Clin Oncol.

[36] Mandrekar JN (2010). Receiver operating characteristic curve in diagnostic test assessment. J Thorac Oncol.

[37] Alonzo TA (2009). Clinical prediction models: a practical approach to development, validation, and updating. Am J Epidemiol.

[38] LeDell E, Petersen M, van der Laan M (2015). Computationally efficient confidence intervals for cross-validated area under the ROC curve estimates. Electron J Stat.

[39] Yang L, Zheng R, Wang N, Yuan Y, Liu S, Li H (2018). Incidence and mortality of stomach cancer in China, 2014. Chin J Cancer Res.

[40] WHO CVD Risk Chart Working Group. World Health Organization cardiovascular disease risk charts: revised models to estimate risk in 21 global regions. Lancet Glob Health. 2019;7:e1332–45. 10.1016/S2214-109X(19)30318-3 PMC7025029 31488387

[41] Tammemägi MC, Katki HA, Hocking WG, Church TR, Caporaso N, Kvale PA (2013). Selection criteria for lung-cancer screening. N Engl J Med.

[42] Yang L, Kartsonaki C, Yao P, de Martel C, Plummer M, Chapman D (2021). The relative and attributable risks of cardia and non-cardia gastric cancer associated with Helicobacter pylori infection in China: a case-cohort study. Lancet Public Health.

[43] Kim GH, Liang PS, Bang SJ, Hwang JH (2016). Screening and surveillance for gastric cancer in the United States: is it needed?. Gastrointest Endosc.

