# Supplementary material for: Development, validation, and evaluation of a risk assessment tool for personalized screening of gastric cancer in Chinese populations
Source: BMC Med. 2023 Apr 27;21:159. doi: 10.1186/s12916-023-02864-0 (PMC10142220; doi:10.1186/s12916-023-02864-0)
Supplement: Supplementary file 1 — Additional file 1: Appendix 1.0. Study design and subjects. Appendix 2.0. Assessment of risk factors. Appendix 3.0. Definition of GC cases. Table S1. Results of univariate Cox regression analysis in the CKB cohort. Table S2. Results of multivariate Cox regression model and corresponding risk points in sensitivity analysis 1: excluding weak variables in the simplified model. Table S3. Results of multivariate Cox regression model and corresponding risk points in sensitivity analysis 2: integrating lifestyle factors as an index. Table S4. Results of multivariate Cox regression model and corresponding risk points in sensitivity analysis 3: excluding participants who had GC diagnosis within the first year after recruitment. Table S5. Results of multivariate Cox regression model and corresponding risk points in sensitivity analysis 4: excluding participants who had cancer at baseline. Table S6. Results and corresponding risk points in sensitivity analysis 5: competing risk model by considering death as a competing event. Table S7. Risk categories by deciles of the GCRS in the CKB cohort. Table S8. Internal validation of the GCRS in different regions of CKB. Table S9. Harrell’s C-index of the GCRS from ten-fold cross validation in the CKB cohort. Table S10. Risk categories of the GCRS in the Changzhou cohort. Table S11. Risk categories and associated 3-year, 5-year, and 10-year risk of incident GC derived from CKB. Table S12. Risk categories of different gastric lesions in the Yangzhou screening program. Table S13. Performance of the GCRS across different predicted risk cutoffs in the Yangzhou screening program. Table S14. Risk categories of different gastric lesions in the Yangzhou screening program in sensitivity analysis 1: excluding weak variables in the simplified model. Table S15. Risk categories of different gastric lesions in the Yangzhou screening program in sensitivity analysis 2: integrating lifestyle factors as an index. Table S16. Risk categories of different [file 12916_2023_2864_MOESM1_ESM.docx]

**Supplementary Materials and Methods**

| **TABLE OF CONTENTS** | | |
| --- | --- | --- |
| **Appendix** |  | **Page** |
| **1.0** | **Study design and subjects** | **3** |
| **2.0** | **Assessment of risk factors** | **3** |
| **3.0** | **Definition of GC cases** | **4** |
| **4.0** | **Tables and Figures** | **6** |
| **Table S1** | **Results of univariate Cox regression analysis in the CKB cohort** | **6** |
| **Table S2** | **Results of multivariate Cox regression model and corresponding risk points in sensitivity analysis 1: excluding weak variables in the simplified model** | **8** |
| **Table S3** | **Results of multivariate Cox regression model and corresponding risk points in sensitivity analysis 2: integrating lifestyle factors as an index** | **9** |
| **Table S4** | **Results of multivariate Cox regression model and corresponding risk points in sensitivity analysis 3: excluding participants who had GC diagnosis within the first year after recruitment** | **10** |
| **Table S5** | **Results of multivariate Cox regression model and corresponding risk points in sensitivity analysis 4: excluding participants who had cancer at baseline** | **12** |
| **Table S6** | **Results and corresponding risk points in sensitivity analysis 5: competing risk model by considering death as a competing event** | **14** |
| **Table S7** | **Risk categories by deciles of the GCRS in the CKB cohort** | **16** |
| **Table S8** | **Internal validation of the GCRS in different regions of CKB** | **17** |
| **Table S9** | **Harrell's C-index of the GCRS from ten-fold cross validation in the CKB cohort** | **18** |
| **Table S10** | **Risk categories of the GCRS in the Changzhou cohort** | **19** |
| **Table S11** | **Risk categories and associated 3-year, 5-year, and 10-year risk of incident GC derived from CKB** | **20** |
| **Table S12** | **Risk categories of different gastric lesions in the Yangzhou screening program** | **21** |
| **Table S13** | **Performance of the GCRS across different predicted risk cutoffs in the Yangzhou screening program** | **22** |
| **Table S14** | **Risk categories of different gastric lesions in the Yangzhou screening program in sensitivity analysis 1: excluding weak variables in the simplified model** | **23** |
| **Table S15** | **Risk categories of different gastric lesions in the Yangzhou screening program in sensitivity analysis 2: integrating lifestyle factors as an index** | **24** |
| **Table S16** | **Risk categories of different gastric lesions in the Yangzhou screening program in sensitivity analysis 3: excluding participants who had GC diagnosis within the first year after recruitment** | **25** |
| **Table S17** | **Risk categories of different gastric lesions in the Yangzhou screening program in sensitivity analysis 4: excluding participants who had cancer at baseline** | **26** |
| **Table S18** | **Risk categories of different gastric lesions in the Yangzhou screening program in sensitivity analysis 5: competing risk model** | **27** |
| **Table S19** | **The GCRS and corresponding 3-year, 5-year, and 10-year risk of incident GC derived from the CKB cohort** | **28** |
| **Fig. S1** | **Study design and eligible participants’ selection procedures in three studies** | **29** |
| **Fig. S2** | **The relationship of the GCRS with incident GC risk in the CKB cohort** | **30** |
| **Fig. S3** | **The relationship of the GCRS with incident GC risk in the Changzhou cohort** | **31** |
| **Fig. S4** | **Calibration and discrimination of the GCRS in sensitivity analysis 1: excluding weak variables in the simplified model** | **32** |
| **Fig. S5** | **Calibration and discrimination of the GCRS in sensitivity analysis 2: integrating lifestyle factors as an index** | **33** |
| **Fig. S6** | **Calibration and discrimination of the GCRS in sensitivity analysis 3: excluding participants who had GC diagnosis within the first year after recruitment** | **34** |
| **Fig. S7** | **Calibration and discrimination of the GCRS in sensitivity analysis 4: excluding participants who had cancer at baseline** | **35** |
| **Fig. S8** | **Calibration and discrimination of the GCRS in sensitivity analysis 5: competing risk model** | **36** |

**1.0 Study design and subjects**

**Development cohort**: The China Kadoorie Biobank (CKB) is the largest prospective cohort study in China of over 0.5 million adults (aged 30-79 years) recruited between June 25, 2004, and July 15, 2008, from ten areas (five urban and five rural) whose study design has been reported previously. Permanent residents of 100-150 rural villages or urban committees were invited to participate in each area. An interviewer-administered electronic questionnaire of each participant was completed and some physical indexes were measured at baseline. All participants provided written informed consent. Regional, national, and international ethics approval was obtained prior to the start of recruitment. For this study, a total of 512,714 adults were initially enrolled. After the exclusion of 264 participants who were prediagnosed with gastric cancer before baseline, 81,047 who were outside the target age range, and 15,060 for whom specific covariates were not available, a total of 416,343 subjects fulfilled all criteria and were included in the final analyses (**Figure S1**). The median follow-up time was 10.1 person-years.

**Validation cohort**: Wujin District is in Changzhou City, located in the southern part of Jiangsu Province on the eastern coast of mainland China. Twenty-three administrative units (villages or street committees) were selected by using the multi-stage cluster sampling method as study areas based on geographic location and levels of economic development. Permanent residents aged 35 years or older were invited by the village doctors. All studies were approved by the relevant institutional review boards and all participants provided written informed consent. A total of 20,803 residents of Wujin District were included in this survey from April 2004 to August 2005 and completed the baseline survey and long-term follow-up. After excluding 42 people with previous gastric cancer, 6,520 people outside the age range of 40-75 years old, and 214 people for whom variable data were not available, 45 people with missing follow-up time, a total of 13,982 people were included for validation, with a median follow-up time of 13.6 person-years.

**Screening program**: The Yangzhou screening program is an ongoing upper gastrointestinal disease screening program for individuals aged 40-75 years old at baseline from Yangzhou City in Jiangsu Province. This is a population-based free-of-charge screening program supported by the local government. This project primarily aimed to identify the risk factors for gastric cancer and precancerous lesions and invited local residents for endoscopy inspection. To encourage participation, individuals who were slightly outside the target age range were not turned away. More than 5,000 people were recruited in Hanjiang District between December 2017 and March 2022. The participants were excluded if they had any current or previous upper gastrointestinal cancers, medical history of surgery or endoscopic resection of the stomach, or health problems that are not suitable for endoscopy, and were unwilling to participate at any stage of the study for any reason. All studies were approved by the relevant institutional review boards and written informed consent was obtained from all participants. Each study participant underwent a face-to-face interview, upper gastrointestinal endoscopy, and pathological biopsy at the baseline survey. A total of 5,718 survey subjects were included, and people outside the age range (n=117) were excluded. Those who lack pathological biopsy reports (n=175) and persons with missing variable data (n=78) were included. A total of 5,348 persons were included for analysis.

**2.0 Assessment of risk factors**

Age, sex, and education level were collected from the baseline questionnaire. Age was divided into 5-year interval groups and education was classified into four levels: illiterate or primary school, middle school, high school, college or above. Physical measurements included weight and height. BMI was calculated as weight in kilograms divided by the square of height in meters and classified into two groups of underweight (BMI <18.5 kg/m^2^) and non-underweight (BMI ≥18.5 kg/m^2^).

In the CKB cohort, questions about tobacco smoking included how often the participants had smoked tobacco at the survey time during the baseline questionnaire. Smoking status was classified as never (smoked <100 cigarettes during lifetime), occasional, former, or regular smoker. Among regular smokers who had stopped smoking due to physical illness were still counted as smokers in the main analyses. Information on the duration (years) of smoking and cigarettes smoked per day for regular smokers and former smokers was also collected. And we calculated pack-years as a measure of the cumulative burden of smoking by the product of the years of smoking (excluding years of quitting smoking) and the number of cigarette packs (the number of cigarettes divided by 20) smoked per day. So total tobacco consumption was categorized as never smoking (0 pack-year), light smoking (<20 pack-years), and heavy smoking (≥20 pack-years).

The detailed questionnaire on alcohol consumption included the frequency of drinking alcohol during the previous 12 months and those who had drunk weekly were asked about the frequency of drinking per week, type of beverage, and amount of intake. Assessment of total pure alcohol consumption in a typical week has been described elsewhere which was calculated as the amount drunk multiplied by frequency (days per week) based on the beverage type, assuming the following alcohol content by volume (v/v): beer 4%, grape wine 12%, rice wine 15%, weak spirits 38%, and strong spirits 53%. In this study, we calculated the average daily alcohol consumption for participants and divided them into two groups: never or light drinkers (<25 g/d in men and <15 g/d in women), excessive drinkers (≥25 g/d in men and ≥15 g/d in women) according to the Chinese Dietary Guidelines 2016 (CDG-2016).

Respondents were also asked about the frequency of habitual dietary consumption during the previous 12 months and five categories of frequency (daily, 4-6 days per week, 1-3 days per week, monthly, or never or rarely) can be chosen. Frequent intake of fresh vegetables and fruits was defined as eating vegetables every day and fruits ≥4 days per week, or eating fruits every day and vegetables ≥4 days per week, otherwise was occasional; while frequent intake of salty foods was defined as eating preserved salty vegetables ≥4 days per week, otherwise was occasional. Physical activity was categorized as active level (≥9.81 MET-h/d) and inactive level (<9.81 MET-h/d).

Personal diagnoses of cancer or peptic ulcer were all self-reported. The first-degree family members’ cancer history including fathers, mothers, and siblings had been collected for each participant. Those who reported at least one first-degree relative had cancer were classified as having a family history of cancer.

Due to different baseline questionnaire settings, some risk factors in the Changzhou cohort and Yangzhou screening program were not defined the same as CKB. For alcohol consumption, the Changzhou and Yangzhou studies did not distinguish between weak spirits and strong spirits, so the average alcohol content of weak spirits and strong spirits in CKB was used instead; the Changzhou cohort did not distinguish between rice wine and grape wine, so the average alcohol content of rice wine and grape wine in CKB was used instead. Vegetable intake in the Changzhou cohort was evaluated by amount rather than frequency. We defined high intake as eating vegetables every day and moderate intake as eating ≥3 days per week. Both in the Changzhou cohort and Yangzhou screening program, frequent consumption of fresh fruits and vegetables was defined as eating vegetables every day and fruits at least 3 days per week, or eating fruits every day and vegetables at least 3 days per week; while low consumption of salty food was defined as eating preserved salty vegetables less than 3 days per week.

**3.0 Definition of GC cases**

In the CKB cohort, all the participants were followed up semi-annually for cancer events from the time they enrolled in the study through the linkage with established chronic disease registries, and the disease surveillance points system in the study areas, as well as the Chinese National Health Insurance claim database. Annual active follow-up was performed by checking against local residential and health insurance records in case of participants who were not linked to the local health insurance database and was confirmed by street committees or village administrators. All outcomes were coded according to the International Classification of Diseases 10th Revision (ICD-10) by trained staff who were masked to baseline information. The outcome in the final analysis was gastric cancer (C16) and medical records were retrieved by December 31, 2016.

The participants in the Changzhou cohort were followed up actively through follow-up questionnaires and disease registries by staff from Changzhou CDC. Information on non-fatal adverse health events will be obtained for certain major categories of disease (such as cancer, cerebrovascular diseases, and cardiovascular diseases) through the established registry systems available and further confirmed by reviewing local medical records or visiting village doctors. For any suspected cases of non-fatal cancer, further confirmation about their diagnosis will be sought by reviewing hospital or other medical records, or by visiting doctors at street or village health clinics to improve the statistical power of the study. Lost participants in each of the active follow-ups were further matched with the local chronic disease registries, cancer registration database, and death surveillance database. The Changzhou cohort completed three follow-up investigations in 2008-2009, 2012-2013, and 2018-2019, respectively.

All participants in the Yangzhou screening program underwent gastroscopy and histological examination during the baseline enrollment. All endoscopic examinations and therapies were performed by well-trained doctors from the endoscopy center of the Affiliated Hospital of Yangzhou University using high-quality white light endoscopes according to the guidelines for cancer screening and early diagnosis and treatment in China, and detailed photographic documentation was taken for each participant accordingly. For participants with undecided healthy or benign lesions, at least two biopsies were taken from the body, antrum, or angulus for histologic examination, while for participants with suspected invasive carcinoma, biopsies were taken from the lesion location and adjacent sites. For this study, all histological specimens were sent to one histopathological laboratory, and each biopsy sample was independently evaluated by two pathologists who were unaware of the endoscopic findings for the diagnosis. Participants with multiple lesions were categorized as having the most severe lesion. The primary outcome in the screening program was histopathologically diagnosed GC, and the secondary outcomes included dysplasia (DYS), intestinal metaplasia (IM), atrophic gastritis (AG), and chronic superficial gastritis (SG). All the diagnoses were based on the gastric epithelial neoplasia classification system from the Japanese Research Society for Gastric Cancer (JRSGC). All participating endoscopists and pathologists were uniformly trained and analyzed according to standard operating procedures.

**4.0 Tables and Figures**

**Table S1. Results of univariate Cox regression analysis in the CKB cohort**

| **Variables** | | **Regression coefficient** | **HR (95% CI) ^a^** | ***P-*value** |
| --- | --- | --- | --- | --- |
| Age at baseline, years | | | | |
|  | 40-44 |  | Reference |  |
|  | 45-49 | 0.54 | 1.72 (1.41 to 2.10) | <0.001 |
|  | 50-54 | 1.02 | 2.77 (2.32 to 3.30) | <0.001 |
|  | 55-59 | 1.50 | 4.49 (3.78 to 5.32) | <0.001 |
|  | 60-64 | 1.85 | 6.36 (5.35 to 7.55) | <0.001 |
|  | 65-69 | 2.09 | 8.08 (6.80 to 9.60) | <0.001 |
|  | 70-75 | 2.33 | 10.32 (8.66 to 12.31) | <0.001 |
| Sex | | | | |
|  | Women |  | Reference |  |
|  | Men | 1.08 | 2.94 (2.73 to 3.17) | <0.001 |
| Education | | | | |
|  | College or above |  | Reference |  |
|  | High school | 0.01 | 1.01 (0.81 to 1.26) | 0.938 |
|  | Middle school | 0.22 | 1.25 (1.02 to 1.54) | 0.035 |
|  | Illiterate or primary school | 0.64 | 1.89 (1.55 to 2.31) | <0.001 |
| BMI ^b^ | | | | |
|  | ≥18.5 |  | Reference |  |
|  | <18.5 | 0.53 | 1.71 (1.48 to 1.97) | <0.001 |
| Pack-years of smoking ^c^ | | | | |
|  | Never (0 pack-year) |  | Reference |  |
|  | >0 to <20 pack-years | 0.73 | 2.08 (1.89 to 2.30) | <0.001 |
|  | ≥20 pack-years | 1.05 | 2.86 (2.65 to 3.10) | <0.001 |
| Alcohol drinking per day ^d^ | | | | |
|  | Never or light |  | Reference |  |
|  | Moderate or heavy | 0.69 | 1.99 (1.80 to 2.19) | <0.001 |
| Intake of fresh vegetables and fruits ^e^ | | | | |
|  | Frequent |  | Reference |  |
|  | Occasional | 0.27 | 1.32 (1.21 to 1.43) | <0.001 |
| Intake of salty foods ^f^ | | | | |
|  | Occasional |  | Reference |  |
|  | Frequent | 0.42 | 1.53 (1.42 to 1.65) | <0.001 |
| Previous cancer diagnosis | | | | |
|  | No |  | Reference |  |
|  | Yes | 1.24 | 3.47 (2.60 to 4.63) | <0.001 |
| Family history of cancer in first-degree relatives | | | | |
|  | No |  | Reference |  |
|  | Yes | 0.39 | 1.48 (1.37 to 1.61) | <0.001 |
| History of peptic ulcer | | | | |
|  | No |  | Reference |  |
|  | Yes | 0.70 | 2.02 (1.77 to 2.30) | <0.001 |
| History of diabetes | | | | |
|  | No |  | Reference |  |
|  | Yes | 0.05 | 1.05 (0.87 to 1.28) | 0.604 |
| Physical activity | | | | |
|  | ≥9.81 MET-h/d |  | Reference |  |
|  | <9.81 MET-h/d | 0.47 | 1.61 (1.49 to 1.73) | <0.001 |

^a^ HR, hazard ratio. CI, confidence interval.

^b^ BMI was calculated as weight in kilograms divided by the square of height in meters. BMI, body mass index.

^c^ Pack-year was calculated by the product of the years of smoking (excluding years of quitting smoking) and the number of cigarette packs (the number of cigarettes divided by 20) smoked per day.

^d^ Never or light alcohol drinking was defined as alcohol intake less than 25 g/day in men and 15 g/day in women in the past year, otherwise was moderate or heavy alcohol drinking.

^e^ Frequent intake of fresh vegetables and fruits was defined as eating vegetables every day and fruits ≥4 days per week or eating fruits every day and vegetables ≥4 days per week, otherwise was occasional.

^f^ Frequent intake of salty foods was defined as eating preserved salty vegetables ≥4 days per week, otherwise was occasional.

**Table S2. Results of multivariate Cox regression model and corresponding risk points in sensitivity analysis 1: excluding weak variables in the simplified model ^a^**

| **Variables** | | **Cases/Person-years** | **Regression coefficient** | **HR (95% CI) ^b^** | | ***P-*value** | **Points**  **assigned** |
| --- | --- | --- | --- | --- | --- | --- | --- |
| Age at baseline, years | | | | | | | |
|  | 40-44 | 168/845,021 |  | Reference |  | | 0 |
|  | 45-49 | 236/687,811 | 0.47 | 1.61 (1.32 to 1.96) | <0.001 | | 1.2 |
|  | 50-54 | 473/860,063 | 0.87 | 2.39 (2.00 to 2.86) | <0.001 | | 2.2 |
|  | 55-59 | 593/666,252 | 1.33 | 3.77 (3.16 to 4.49) | <0.001 | | 3.3 |
|  | 60-64 | 572/454,473 | 1.66 | 5.25 (4.41 to 6.26) | <0.001 | | 4.1 |
|  | 65-69 | 570/357,890 | 1.89 | 6.65 (5.58 to 7.93) | <0.001 | | 4.7 |
|  | 70-75 | 477/236,230 | 2.11 | 8.29 (6.92 to 9.93) | <0.001 | | 5.3 |
| Sex | | | | | | | |
|  | Women | 1,033/2,447,930 |  | Reference |  | | 0 |
|  | Men | 2,056/1,659,810 | 1.06 | 2.89 (2.68 to 3.12) | <0.001 | | 2.6 |
| Education | | | | | | | |
|  | College or above | 102/209,414 |  | Reference |  | | 0 |
|  | High school | 310/630,316 | 0.45 | 1.57 (1.26 to 1.97) | <0.001 | | 1.1 |
|  | Middle school | 664/1,088,309 | 0.62 | 1.86 (1.51 to 2.30) | <0.001 | | 1.6 |
|  | Illiterate or primary school | 2,013/2,179,701 | 0.79 | 2.20 (1.80 to 2.69) | <0.001 | | 2.0 |
| Intake of salty foods ^c^ | | | | | | | |
|  | Occasional | 2,105/3,146,493 |  | Reference |  | | 0 |
|  | Frequent | 984/961,247 | 0.41 | 1.51 (1.40 to 1.63) | <0.001 | | 1.0 |
| Previous cancer diagnosis | | | | | | | |
|  | No | 3,042/4,089,431 |  | Reference |  | | 0 |
|  | Yes | 47/18,309 | 1.08 | 2.96 (2.22 to 3.95) | <0.001 | | 2.7 |
| Family history of cancer in first-degree relatives | | | | | | | |
|  | No | 2,328/3,366,039 |  | Reference |  | | 0 |
|  | Yes | 761/741,701 | 0.40 | 1.49 (1.38 to 1.62) | <0.001 | | 1.0 |
| History of peptic ulcer | | | | | | | |
|  | No | 2,844/3,939,709 |  | Reference |  | | 0 |
|  | Yes | 245/168,031 | 0.48 | 1.61 (1.41 to 1.84) | <0.001 | | 1.2 |

^a^ The simplified model was created based on a subset of strong predictors for gastric cancer (assigned points ≥4.0; including age, sex, education level, intake of salty foods, family history of cancer in first-degree relatives, personal medical history of cancer and peptic ulcer).

^b^ HR, hazard ratio. CI, confidence.

^c^ Frequent intake of salty foods was defined as eating preserved salty vegetables ≥4 days per week, otherwise was occasional.

**Table S3. Results of multivariate Cox regression model and corresponding risk points in sensitivity analysis 2: integrating lifestyle factors as an index ^a^**

| **Variables** | | **Cases/Person-years** | **Regression coefficient** | **HR (95% CI) ^b^** | ***P-*value** | **Points**  **assigned** |
| --- | --- | --- | --- | --- | --- | --- |
| Age at baseline, years | | | | | | |
|  | 40-44 | 168/845,021 |  | Reference |  | 0 |
|  | 45-49 | 236/687,811 | 0.47 | 1.60 (1.31 to 1.95) | <0.001 | 1.4 |
|  | 50-54 | 473/860,063 | 0.88 | 2.42 (2.02 to 2.89) | <0.001 | 2.6 |
|  | 55-59 | 593/666,252 | 1.34 | 3.81 (3.20 to 4.54) | <0.001 | 3.9 |
|  | 60-64 | 572/454,473 | 1.67 | 5.33 (4.47 to 6.35) | <0.001 | 4.9 |
|  | 65-69 | 570/357,890 | 1.92 | 6.80 (5.69 to 8.11) | <0.001 | 5.6 |
|  | 70-75 | 477/236,230 | 2.14 | 8.52 (7.10 to 10.21) | <0.001 | 6.3 |
| Sex | | | | | | |
|  | Women | 1,033/2,447,930 |  | Reference |  | 0 |
|  | Men | 2,056/1,659,810 | 0.91 | 2.48 (2.28 to 2.69) | <0.001 | 2.7 |
| Education | | | | | | |
|  | College or above | 102/209,414 |  | Reference |  | 0 |
|  | High school | 310/630,316 | 0.38 | 1.46 (1.17 to 1.83) | 0.001 | 1.1 |
|  | Middle school | 664/1,088,309 | 0.51 | 1.67 (1.35 to 2.06) | <0.001 | 1.5 |
|  | Illiterate or primary school | 2,013/2,179,701 | 0.62 | 1.87 (1.52 to 2.29) | <0.001 | 1.8 |
| Lifestyle categories ^c^ | | | | | | |
|  | Favorable | 256/666,672 |  | Reference |  | 0 |
|  | Intermediate | 2,203/3,077,761 | 0.34 | 1.41 (1.23 to 1.61) | <0.001 | 1.0 |
|  | Unfavorable | 630/363,307 | 0.76 | 2.13 (1.82 to 2.49) | <0.001 | 2.2 |
| Previous cancer diagnosis | | | | | | |
|  | No | 3,042/4,089,431 |  | Reference |  | 0 |
|  | Yes | 47/18,309 | 1.08 | 2.96 (2.21 to 3.95) | <0.001 | 3.2 |
| Family history of cancer in first-degree relatives | | | | | | |
|  | No | 2,328/3,366,039 |  | Reference |  | 0 |
|  | Yes | 761/741,701 | 0.42 | 1.52 (1.40 to 1.65) | <0.001 | 1.2 |
| History of peptic ulcer | | | | | | |
|  | No | 2,844/3,939,709 |  | Reference |  | 0 |
|  | Yes | 245/168,031 | 0.48 | 1.61 (1.42 to 1.84) | <0.001 | 1.4 |

^a^ The healthy lifestyle index was generated by integrating five modifiable lifestyle factors, i.e., BMI, smoking, alcohol use, consumption of fresh vegetables and fruits, and salty food intake.

^b^ HR, hazard ratio. CI, confidence interval.

^c^ Unhealthy lifestyle factors were defined as (i) smoking 20 pack-years or more at recruitment, (ii) moderate or high consumption of alcohol (≥15 g/d for women and ≥25 g/d for men) in accordance with the CDG-2016 guidelines, (iii) BMI <18.5 kg/m^2^, (iv) occasional consumption of fresh vegetables and fruits and (v) frequent intake of salty foods. Participants were divided into favorable (0 unhealthy lifestyle factors), intermediate (1 or 2 unhealthy lifestyle factors), or unfavorable (≥3 unhealthy lifestyle factors) according to the number of unhealthy lifestyle factors.

**Table S4. Results of multivariate Cox regression model and corresponding risk points in sensitivity analysis 3: excluding participants who had GC diagnosis within the first year after recruitment**

| **Variables** | | **Cases/Person-years** | **Regression coefficient** | **HR (95% CI) ^a^** | ***P*-value** | **Points assigned** |
| --- | --- | --- | --- | --- | --- | --- |
| Age at baseline, years | | | | | | |
|  | 40-44 | 159/845,015 |  | Reference |  | 0 |
|  | 45-49 | 219/687,803 | 0.45 | 1.57 (1.28 to 1.92) | <0.001 | 4.3 |
|  | 50-54 | 430/860,045 | 0.84 | 2.33 (1.94 to 2.80) | <0.001 | 8.1 |
|  | 55-59 | 545/666,233 | 1.32 | 3.76 (3.14 to 4.50) | <0.001 | 12.7 |
|  | 60-64 | 515/454,441 | 1.64 | 5.16 (4.30 to 6.19) | <0.001 | 15.8 |
|  | 65-69 | 518/357,865 | 1.90 | 6.69 (5.57 to 8.04) | <0.001 | 18.3 |
|  | 70-75 | 418/236,203 | 2.11 | 8.21 (6.80 to 9.92) | <0.001 | 20.2 |
| Sex | | | | | | |
|  | Women | 949/2,447,893 |  | Reference |  | 0 |
|  | Men | 1,855/1,659,711 | 0.84 | 2.32 (2.08 to 2.60) | <0.001 | 8.1 |
| Education | | | | | | |
|  | College or above | 94/209,409 |  | Reference |  | 0 |
|  | High school | 285/630,303 | 0.39 | 1.48 (1.17 to 1.87) | 0.001 | 3.8 |
|  | Middle school | 617/1,088,287 | 0.54 | 1.72 (1.38 to 2.14) | <0.001 | 5.2 |
|  | Illiterate or primary school | 1,808/2,179,604 | 0.64 | 1.89 (1.52 to 2.34) | <0.001 | 6.1 |
| BMI ^b^ | | | | | | |
|  | ≥18.5 | 2,643/3,943,447 |  | Reference |  | 0 |
|  | <18.5 | 161/164,157 | 0.12 | 1.13 (0.96 to 1.33) | 0.140 | 1.2 |
| Pack-years of smoking ^c^ | | | | | | |
|  | Never (0 pack-year) | 1,265/2,772,868 |  | Reference |  | 0 |
|  | >0 to <20 pack-years | 511/536,544 | 0.14 | 1.15 (1.02 to 1.30) | 0.026 | 1.3 |
|  | ≥20 pack-years | 1,028/798,192 | 0.23 | 1.26 (1.12 to 1.41) | <0.001 | 2.2 |
| Alcohol drinking per day ^d^ | | | | | | |
|  | Never or light | 2,345/3,750,421 |  | Reference |  | 0 |
|  | Moderate or heavy | 459/357,183 | 0.21 | 1.23 (1.11 to 1.37) | <0.001 | 2.0 |
| Intake of fresh vegetables and fruits ^e^ | | | | | | |
|  | Frequent | 636/1,134,003 |  | Reference |  | 0 |
|  | Occasional | 2,168/2,973,601 | 0.10 | 1.11 (1.01 to 1.22) | 0.029 | 1.0 |
| Intake of salty foods ^f^ | | | | | | |
|  | Occasional | 1,903/3,146,394 |  | Reference |  | 0 |
|  | Frequent | 901/961,209 | 0.41 | 1.50 (1.38 to 1.63) | <0.001 | 3.9 |
| Previous cancer diagnosis | | | | | | |
|  | No | 2,765/4,089,296 |  | Reference |  | 0 |
|  | Yes | 39/18,307 | 1.02 | 2.76 (2.01 to 3.79) | <0.001 | 9.8 |
| Family history of cancer in first-degree relatives | | | | | | |
|  | No | 2,098/3,365,929 |  | Reference |  | 0 |
|  | Yes | 706/741,675 | 0.43 | 1.54 (1.41 to 1.68) | <0.001 | 4.1 |
| History of peptic ulcer | | | | | | |
|  | No | 2,605/3,939,592 |  | Reference |  | 0 |
|  | Yes | 199/168,011 | 0.35 | 1.42 (1.23 to 1.64) | <0.001 | 3.4 |

^a^ HR, hazard ratio. CI, confidence interval.

^b^ BMI was calculated as weight in kilograms divided by the square of height in meters. BMI, body mass index. BMI was included after stepwise regression analysis.

^c^ Pack-year was calculated by the product of the years of smoking (excluding years of quitting smoking) and the number of cigarette packs (the number of cigarettes divided by 20) smoked per day.

^d^ Never or light alcohol drinking was defined as alcohol intake less than 25 g/day in men and 15 g/day in women in the past year, otherwise was moderate or heavy alcohol drinking.

^e^ Frequent intake of fresh vegetables and fruits was defined as eating vegetables every day and fruits ≥4 days per week or eating fruits every day and vegetables ≥4 days per week, otherwise was occasional.

^f^ Frequent intake of salty foods was defined as eating preserved salty vegetables ≥4 days per week, otherwise was occasional.

**Table S5. Results of multivariate Cox regression model and corresponding risk points in sensitivity analysis 4: excluding participants who had cancer at baseline**

| **Variables** | | **Cases/Person-years** | **Regression coefficient** | **HR (95% CI) ^a^** | ***P*-value** | **Points assigned** |
| --- | --- | --- | --- | --- | --- | --- |
| Age at baseline, years | | | | | | |
|  | 40-44 | 168/843,593 |  | Reference |  | 0 |
|  | 45-49 | 234/685,533 | 0.46 | 1.59 (1.30 to 1.93) | <0.001 | 4.5 |
|  | 50-54 | 471/856,251 | 0.88 | 2.40 (2.01 to 2.87) | <0.001 | 8.5 |
|  | 55-59 | 571/662,364 | 1.31 | 3.69 (3.09 to 4.40) | <0.001 | 12.7 |
|  | 60-64 | 569/451,794 | 1.67 | 5.33 (4.47 to 6.36) | <0.001 | 16.2 |
|  | 65-69 | 559/355,517 | 1.90 | 6.69 (5.60 to 7.99) | <0.001 | 18.4 |
|  | 70-75 | 470/234,380 | 2.13 | 8.45 (7.04 to 10.14) | <0.001 | 20.7 |
| Sex | | | | | | |
|  | Women | 1,012/2,435,046 |  | Reference |  | 0 |
|  | Men | 2,030/1,654,385 | 0.88 | 2.42 (2.17 to 2.69) | <0.001 | 8.6 |
| Education | | | | | | |
|  | College or above | 100/207,832 |  | Reference |  | 0 |
|  | High school | 305/627,206 | 0.40 | 1.49 (1.18 to 1.87) | <0.001 | 3.8 |
|  | Middle school | 653/1,084,078 | 0.53 | 1.70 (1.37 to 2.11) | <0.001 | 5.1 |
|  | Illiterate or primary school | 1,984/2,170,316 | 0.66 | 1.93 (1.57 to 2.38) | <0.001 | 6.4 |
| BMI ^b^ | | | | | | |
|  | ≥18.5 | 2,846/3,926,414 |  | Reference |  | 0 |
|  | <18.5 | 196/163,017 | 0.23 | 1.26 (1.09 to 1.46) | 0.002 | 2.3 |
| Pack-years of smoking ^c^ | | | | | | |
|  | Never (0 pack-year) | 1,369/2,759,128 |  | Reference |  | 0 |
|  | >0 to <20 pack-years | 549/534,850 | 0.10 | 1.11 (0.98 to 1.25) | 0.088 | 1.0 |
|  | ≥20 pack-years | 1,124/795,453 | 0.21 | 1.23 (1.10 to 1.37) | <0.001 | 2.0 |
| Alcohol drinking per day ^d^ | | | | | | |
|  | Never or light | 2,552/3,732,728 |  | Reference |  | 0 |
|  | Moderate or heavy | 490/356,703 | 0.18 | 1.20 (1.08 to 1.33) | <0.001 | 1.7 |
| Intake of fresh vegetables and fruits ^e^ | | | | | | |
|  | Frequent | 677/1,127,304 |  | Reference |  | 0 |
|  | Occasional | 2,365/2,962,127 | 0.11 | 1.12 (1.02 to 1.22) | 0.016 | 1.1 |
| Intake of salty foods ^f^ | | | | | | |
|  | Occasional | 2,074/3,132,399 |  | Reference |  | 0 |
|  | Frequent | 968/957,032 | 0.40 | 1.49 (1.38 to 1.61) | <0.001 | 3.9 |
| Family history of cancer in first-degree relatives | | | | | | |
|  | No | 2,297/3,353,329 |  | Reference |  | 0 |
|  | Yes | 745/736,102 | 0.41 | 1.51 (1.39 to 1.64) | <0.001 | 4.0 |
| History of peptic ulcer | | | | | | |
|  | No | 2,799/3,922,174 |  | Reference |  | 0 |
|  | Yes | 243/167,257 | 0.48 | 1.61 (1.41 to 1.84) | <0.001 | 4.6 |

^a^ HR, hazard ratio. CI, confidence interval.

^b^ BMI was calculated as weight in kilograms divided by the square of height in meters. BMI, body mass index.

^c^ Pack-year was calculated by the product of the years of smoking (excluding years of quitting smoking) and the number of cigarette packs (the number of cigarettes divided by 20) smoked per day.

^d^ Never or light alcohol drinking was defined as alcohol intake less than 25 g/day in men and 15 g/day in women in the past year, otherwise was moderate or heavy alcohol drinking.

^e^ Frequent intake of fresh vegetables and fruits was defined as eating vegetables every day and fruits ≥4 days per week or eating fruits every day and vegetables ≥4 days per week, otherwise was occasional.

^f^ Frequent intake of salty foods was defined as eating preserved salty vegetables ≥4 days per week, otherwise was occasional.

**Table S6. Results and corresponding risk points in sensitivity analysis 5: competing risk model by considering death as a competing event**

| **Variables** | | **Cases/Person-years** | **Regression coefficient** | **HR (95% CI) ^a^** | ***P*-value** | **Points assigned** |
| --- | --- | --- | --- | --- | --- | --- |
| Age at baseline, years | | | | | | |
|  | 40-44 | 168/845,021 |  | Reference |  | 0 |
|  | 45-49 | 236/687,811 | 0.47 | 1.59 (1.31 to 1.94) | <0.001 | 4.7 |
|  | 50-54 | 473/860,063 | 0.87 | 2.39 (2.00 to 2.86) | <0.001 | 8.8 |
|  | 55-59 | 593/666,252 | 1.33 | 3.79 (3.18 to 4.51) | <0.001 | 13.4 |
|  | 60-64 | 572/454,473 | 1.66 | 5.26 (4.42 to 6.27) | <0.001 | 16.7 |
|  | 65-69 | 570/357,890 | 1.89 | 6.65 (5.57 to 7.93) | <0.001 | 19.1 |
|  | 70-75 | 477/236,230 | 2.11 | 8.28 (6.90 to 9.93) | <0.001 | 21.3 |
| Sex | | | | | | |
|  | Women | 1,033/2,447,930 |  | Reference |  | 0 |
|  | Men | 2,056/1,659,810 | 0.87 | 2.39 (2.15 to 2.65) | <0.001 | 8.8 |
| Education | | | | | | |
|  | College or above | 102/209,414 |  | Reference |  | 0 |
|  | High school | 310/630,316 | 0.40 | 1.49 (1.19 to 1.87) | <0.001 | 4.0 |
|  | Middle school | 664/1,088,309 | 0.54 | 1.71 (1.38 to 2.12) | <0.001 | 5.4 |
|  | Illiterate or primary school | 2,013/2,179,701 | 0.66 | 1.94 (1.58 to 2.38) | <0.001 | 6.7 |
| BMI ^b^ | | | | | | |
|  | ≥18.5 | 2,885/3,943,563 |  | Reference |  | 0 |
|  | <18.5 | 204/164,177 | 0.24 | 1.27 (1.10 to 1.47) | 0.001 | 2.4 |
| Pack-years of smoking ^c^ | | | | | | |
|  | Never (0 pack-year) | 1,389/2,772,929 |  | Reference |  | 0 |
|  | >0 to <20 pack-years | 559/536,569 | 0.12 | 1.12 (1.00 to 1.26) | 0.051 | 1.2 |
|  | ≥20 pack-years | 1,141/798,243 | 0.22 | 1.24 (1.12 to 1.38) | <0.001 | 2.2 |
| Alcohol drinking per day ^d^ | | | | | | |
|  | Never or light | 2,598/3,750,542 |  | Reference |  | 0 |
|  | Moderate or heavy | 491/357,198 | 0.17 | 1.19 (1.07 to 1.32) | <0.001 | 1.7 |
| Intake of fresh vegetables and fruits ^e^ | | | | | | |
|  | Frequent | 695/1,134,030 |  | Reference |  | 0 |
|  | Occasional | 2,394/2,973,710 | 0.10 | 1.10 (1.01 to 1.21) | 0.030 | 1.0 |
| Intake of salty foods ^f^ | | | | | | |
|  | Occasional | 2,105/3,146,493 |  | Reference |  | 0 |
|  | Frequent | 984/961,247.4 | 0.40 | 1.50 (1.39 to 1.62) | <0.001 | 4.1 |
| Previous cancer diagnosis | | | | | | |
|  | No | 3,042/4,089,431 |  | Reference |  | 0 |
|  | Yes | 47/18,309 | 1.06 | 2.89 (2.16 to 3.86) | <0.001 | 10.7 |
| Family history of cancer in first-degree relatives | | | | | | |
|  | No | 2,328/3,366,039 |  | Reference |  | 0 |
|  | Yes | 761/741,701 | 0.41 | 1.50 (1.38 to 1.63) | <0.001 | 4.1 |
| History of peptic ulcer | | | | | | |
|  | No | 2,844/3,939,709 |  | Reference |  | 0 |
|  | Yes | 245/168,031 | 0.47 | 1.60 (1.0 to 1.82) | <0.001 | 4.7 |

^a^ HR, hazard ratio. CI, confidence interval.

^b^ BMI was calculated as weight in kilograms divided by the square of height in meters. BMI, body mass index.

^c^ Pack-year was calculated by the product of the years of smoking (excluding years of quitting smoking) and the number of cigarette packs (the number of cigarettes divided by 20) smoked per day.

^d^ Never or light alcohol drinking was defined as alcohol intake less than 25 g/day in men and 15 g/day in women in the past year, otherwise was moderate or heavy alcohol drinking.

^e^ Frequent intake of fresh vegetables and fruits was defined as eating vegetables every day and fruits ≥4 days per week or eating fruits every day and vegetables ≥4 days per week, otherwise was occasional.

^f^ Frequent intake of salty foods was defined as eating preserved salty vegetables ≥4 days per week, otherwise was occasional.

**Table S7. Risk categories by deciles of the GCRS in the CKB cohort**

| **Risk category ^a^** | **GCRS range** | **Total (n=416,343)**  **No.** | **Person-years** | **Incident gastric cancer (n=3,089)** | | | |
| --- | --- | --- | --- | --- | --- | --- | --- |
|  |  |  |  | **No.** | **Incidence rate (per 100,000 person-years)** | **Proportion ^b^**  **(%)** | **Cumulative proportion ^c^ (%)** |
| D10 | 35.3-62.2 | 41,140 | 367,569 | 1,059 | 288.11 | 34.28 | 34.28 |
| D9 | 30.7-35.2 | 41,975 | 399,418 | 584 | 146.21 | 18.91 | 53.19 |
| D8 | 27.7-30.6 | 41,468 | 401,610 | 383 | 95.37 | 12.40 | 65.59 |
| D7 | 24.9-27.6 | 35,524 | 347,398 | 254 | 73.12 | 8.22 | 73.81 |
| D6 | 22.0-24.8 | 48,053 | 478,842 | 296 | 61.82 | 9.58 | 83.39 |
| D5 | 19.9-21.9 | 38,543 | 388,197 | 154 | 39.67 | 4.99 | 88.38 |
| D4 | 16.3-19.8 | 36,873 | 371,629 | 104 | 27.98 | 3.37 | 91.74 |
| D3 | 13.7-16.2 | 45,875 | 468,174 | 121 | 25.85 | 3.92 | 95.66 |
| D2 | 9.1-13.6 | 44,222 | 450,814 | 76 | 16.86 | 2.46 | 98.12 |
| D1 | 0.0-9.0 | 42,670 | 434,090 | 58 | 13.36 | 1.88 | 100.00 |

^a^ Participants in the CKB cohort were divided into ten equal groups according to the GCRS. GCRS, gastric cancer risk score.

^b^ The proportion was calculated by dividing the number of incident gastric cancer cases in each risk category by the total number of GC cases.

^c^ Cumulative proportion was calculated by dividing the number of incident gastric cancer cases accumulated to this category by the total number of GC cases.

**Table S8. Internal validation of the GCRS in different regions of CKB**

| **Variable** | | **CKB** | | | | |
| --- | --- | --- | --- | --- | --- | --- |
|  |  | **Cases/Total** | **HR** | **95% CI** | ***P*-value** | **Harrell's C (95% CI)** |
| Full cohort | | 3,089/416,343 | 1.11 | 1.10-1.11 | <0.001 | 0.754 (0.745-0.762) |
| Region | |  |  |  |  |  |
|  | Rural | 1,546/228,453 | 1.11 | 1.10-1.11 | <0.001 | 0.753 (0.741-0.765) |
|  | Urban | 1,543/187,890 | 1.11 | 1.10-1.11 | <0.001 | 0.754 (0.742-0.766) |
| Study region | |  |  |  |  |  |
|  | Qingdao (Urban) | 309/29,344 | 1.09 | 1.08-1.11 | <0.001 | 0.736 (0.708-0.764) |
|  | Harbin (Urban) | 280/47,916 | 1.11 | 1.09-1.12 | <0.001 | 0.763 (0.736-0.790) |
|  | Haikou (Urban) | 140/23,360 | 1.09 | 1.07-1.11 | <0.001 | 0.710 (0.668-0.751) |
|  | Suzhou (Urban) | 644/44,256 | 1.11 | 1.10-1.12 | <0.001 | 0.756 (0.737-0.774) |
|  | Liuzhou (Urban) | 170/43,014 | 1.08 | 1.06-1.10 | <0.001 | 0.695 (0.655-0.735) |
|  | Sichuan (Rural) | 364/44,354 | 1.10 | 1.09-1.11 | <0.001 | 0.747 (0.721-0.772) |
|  | Gansu (Rural) | 348/35,884 | 1.11 | 1.09-1.12 | <0.001 | 0.753 (0.728-0.778) |
|  | Henan (Rural) | 484/50,246 | 1.12 | 1.10-1.13 | <0.001 | 0.774 (0.754-0.794) |
|  | Zhejiang (Rural) | 285/49,239 | 1.11 | 1.09-1.12 | <0.001 | 0.755 (0.726-0.783) |
|  | Hunan (Rural) | 65/48,730 | 1.08 | 1.05-1.11 | <0.001 | 0.681 (0.605-0.757) |

**Table S9. Harrell's C-index of the GCRS from ten-fold cross validation in the CKB cohort**

| **Ten-fold cross validation test sets** | **GCRS** | **Excluding weak variables in the simplified model ^a^** | **Integrating lifestyle factors as an index ^b^** | **Excluding GC participants diagnosed within the first year after recruitment** | **Excluding cancer patients at baseline** | **Competing risk model** |
| --- | --- | --- | --- | --- | --- | --- |
| 1 | 0.747 | 0.746 | 0.746 | 0.729 | 0.738 | 0.747 |
| 2 | 0.739 | 0.739 | 0.737 | 0.764 | 0.752 | 0.739 |
| 3 | 0.743 | 0.743 | 0.740 | 0.744 | 0.752 | 0.743 |
| 4 | 0.772 | 0.768 | 0.768 | 0.760 | 0.766 | 0.772 |
| 5 | 0.767 | 0.765 | 0.769 | 0.746 | 0.751 | 0.767 |
| 6 | 0.763 | 0.761 | 0.759 | 0.755 | 0.744 | 0.764 |
| 7 | 0.757 | 0.756 | 0.755 | 0.736 | 0.766 | 0.757 |
| 8 | 0.743 | 0.740 | 0.740 | 0.721 | 0.740 | 0.743 |
| 9 | 0.751 | 0.748 | 0.754 | 0.755 | 0.752 | 0.751 |
| 10 | 0.755 | 0.755 | 0.749 | 0.785 | 0.767 | 0.755 |
| Average C-index | 0.754 | 0.752 | 0.752 | 0.750 | 0.753 | 0.754 |

^a^ The simplified model was created based on a subset of strong predictors for gastric cancer (assigned points ≥4.0; including age, sex, education level, intake of salty foods, family history of cancer in first-degree relatives, personal medical history of cancer and peptic ulcer).

^b^ The healthy lifestyle index was generated by integrating five modifiable lifestyle factors, i.e., BMI, smoking, alcohol use, consumption of fresh vegetables and fruits, and salty food intake.

**Table S10. Risk categories of the GCRS in the Changzhou cohort**

| **GCRS cut-offs ^a^** | **Total (n=13,982)** | | **Person-years** | **Incident gastric cancer (n=329)** | | | |
| --- | --- | --- | --- | --- | --- | --- | --- |
|  | **No.** | **%** |  | **No.** | **Incidence rate (per 100,000 person-years)** | **Proportion ^b^**  **(%)** | **Cumulative proportion ^c^ (%)** |
| 35.3~ | 1,697 | 12.14 | 19,879 | 117 | 588.56 | 35.56 | 35.56 |
| 30.7-35.2 | 1,553 | 11.11 | 19,496 | 54 | 276.99 | 16.41 | 51.98 |
| 27.7-30.6 | 1,688 | 12.07 | 21,568 | 49 | 227.19 | 14.89 | 66.87 |
| 24.9-27.6 | 1,288 | 9.21 | 16,840 | 35 | 207.84 | 10.64 | 77.51 |
| 22.0-24.8 | 1,828 | 13.07 | 24,308 | 29 | 119.30 | 8.81 | 86.32 |
| 19.9-21.9 | 1,145 | 8.19 | 15,348 | 17 | 110.77 | 5.17 | 91.49 |
| 16.3-19.8 | 1,272 | 9.10 | 17,277 | 8 | 46.31 | 2.43 | 93.92 |
| 13.7-16.2 | 1,413 | 10.11 | 19,224 | 8 | 41.62 | 2.43 | 96.35 |
| 9.1-13.6 | 1,291 | 9.23 | 17,596 | 10 | 56.83 | 3.04 | 99.39 |
| ~9.0 | 807 | 5.77 | 11,094 | 2 | 18.03 | 0.61 | 100.00 |

^a^ Participants in the Changzhou cohort were divided into ten groups according to the cut-offs of the GCRS deciles in the CKB cohort. GCRS, gastric cancer risk score.

^b^ The proportion was calculated by dividing the number of incident gastric cancer cases in each risk category by the total number of GC cases.

^c^ Cumulative proportion was calculated by dividing the number of incident gastric cancer cases accumulated to this category by the total number of GC cases.

**Table S11. Risk categories and associated 3-year, 5-year, and 10-year risk of incident GC derived from CKB**

| **Risk category ^a^** | **GCRS range** | **3 years (875/416,343)** | | **5 years (1,408/416,343)** | | **10 years (2,863/416,343)** | |
| --- | --- | --- | --- | --- | --- | --- | --- |
|  |  | **Absolute risk (%)** | **HR (95% CI) ^b^** | **Absolute risk (%)** | **HR (95% CI) ^b^** | **Absolute risk (%)** | **HR (95% CI) ^b^** |
| D1 | 0.0-9.0 | 0.03 | 1 (reference) | 0.04 | 1 (reference) | 0.10 | 1 (reference) |
| D2 | 9.1-13.6 | 0.05 | 1.49 (0.70 to 3.18) | 0.07 | 1.65 (0.90 to 3.00) | 0.17 | 1.30 (0.91 to 1.86) |
| D3 | 13.7-16.2 | 0.07 | 1.86 (0.90 to 3.84) | 0.11 | 2.46 (1.41 to 4.30) | 0.25 | 1.93 (1.39 to 2.69) |
| D4 | 16.3-19.8 | 0.09 | 2.00 (0.95 to 4.20) | 0.15 | 2.79 (1.59 to 4.91) | 0.33 | 2.14 (1.53 to 3.00) |
| D5 | 19.9-21.9 | 0.12 | 4.03 (2.07 to 7.85) | 0.19 | 3.98 (2.32 to 6.80) | 0.42 | 3.11 (2.27 to 4.27) |
| D6 | 22.0-24.8 | 0.16 | 6.38 (3.40 to 11.99) | 0.26 | 6.54 (3.94 to 10.85) | 0.57 | 4.67 (3.47 to 6.29) |
| D7 | 24.9-27.6 | 0.21 | 7.43 (3.93 to 14.05) | 0.34 | 7.22 (4.32 to 12.06) | 0.74 | 5.37 (3.98 to 7.26) |
| D8 | 27.7-30.6 | 0.27 | 9.83 (5.28 to 18.30) | 0.44 | 10.19 (6.19 to 16.78) | 0.98 | 6.75 (5.04 to 9.04) |
| D9 | 30.7-35.2 | 0.40 | 14.44 (7.84 to 26.62) | 0.65 | 15.95 (9.77 to 26.05) | 1.45 | 10.66 (8.02 to 14.17) |
| D10 | 35.3-62.2 | 0.73 | 33.90 (18.61 to 61.77) | 1.20 | 34.02 (21.00 to 55.13) | 2.66 | 20.26 (15.33 to 26.78) |

^a^ Participants in the CKB cohort were divided into ten equal groups according to the GCRS, and the HRs for each group were compared with those in the lowest of the ten groups (HR 1 (reference)) of the risk score. HRs and 95% CIs were derived from the Cox regression model. GCRS, gastric cancer risk score.

^b^ HR, hazard ratio. CI, confidence interval.

**Table S12. Risk categories of different gastric lesions in the Yangzhou screening program**

| **GCRS cut-offs ^a^** | **Total (n=5,348)** | **GC (n=49) ^b^** | | **DYS (n=163)** | | **IM (n=868)** | | **AG (n=1,626)** | | **Normal (n=2,642)** | |
| --- | --- | --- | --- | --- | --- | --- | --- | --- | --- | --- | --- |
|  | **No. (%)** | **No. (%)** | **CP (%) ^c^** | **No. (%)** | **CP (%)** | **No. (%)** | **CP (%)** | **No. (%)** | **CP (%)** | **No. (%)** | **CP (%)** |
| 35.3~ | 893 (16.70) | 31 (3.47) | 63.27 | 41 (4.59) | 25.15 | 203 (22.73) | 23.39 | 223 (24.97) | 13.71 | 395 (44.23) | 14.95 |
| 30.7-35.2 | 652 (12.19) | 9 (1.38) | 81.63 | 34 (5.21) | 46.01 | 116 (17.79) | 36.75 | 166 (25.46) | 23.92 | 327 (50.15) | 27.33 |
| 27.7-30.6 | 713 (13.33) | 2 (0.28) | 85.71 | 24 (3.37) | 60.74 | 132 (18.51) | 51.96 | 215 (30.15) | 37.15 | 340 (47.69) | 40.20 |
| 24.9-27.6 | 582 (10.88) | 4 (0.69) | 93.88 | 11 (1.89) | 67.48 | 92 (15.81) | 62.56 | 176 (30.24) | 47.97 | 299 (51.37) | 51.51 |
| 22.0-24.8 | 685 (12.81) | 1 (0.15) | 95.92 | 15 (2.19) | 76.69 | 117 (17.08) | 76.04 | 217 (31.68) | 61.32 | 335 (48.91) | 64.19 |
| 19.9-21.9 | 335 (6.26) | 1 (0.30) | 97.96 | 6 (1.79) | 80.37 | 39 (11.64) | 80.53 | 122 (36.42) | 68.82 | 167 (49.85) | 70.51 |
| 16.3-19.8 | 450 (8.41) | 1 (0.22) | 100.00 | 13 (2.89) | 88.34 | 62 (13.78) | 87.67 | 157 (34.89) | 78.47 | 217 (48.22) | 78.73 |
| 13.7-16.2 | 555 (10.38) | 0 (0.00) | 100.00 | 11 (1.98) | 95.09 | 72 (12.97) | 95.97 | 179(32.25) | 89.48 | 293 (52.79) | 89.82 |
| 9.1-13.6 | 339 (6.34) | 0 (0.00) | 100.00 | 7 (2.06) | 99.39 | 28 (8.26) | 99.19 | 117 (34.51) | 96.68 | 187 (55.16) | 96.90 |
| ~9.0 | 144 (2.69) | 0 (0.00) | 100.00 | 1 (0.69) | 100.00 | 7 (4.86) | 100.00 | 54 (37.50) | 100.00 | 82 (56.94) | 100.00 |

^a^ Participants in the Yangzhou screening program were divided into divided into ten groups according to the cut-offs of the GCRS deciles in the CKB cohort. GCRS, gastric cancer risk score.

^b^ GC, gastric cancer. DYS, dysplasia. IM, intestinal metaplasia. AG, atrophic gastritis. “Normal” biopsy report includes the diagnosis of chronic superficial gastritis or no lesions.

^c^ Cumulative proportion (CP) was calculated by dividing the number of each lesion accumulated to this category by the total number of each lesion.

**Table S13. Performance of the GCRS across different predicted risk cutoffs in the Yangzhou screening program**

| **GCRS cut-offs ^a^** | **Total (n=5,348)** | **GC (n=49)** | **Percent of high-risk population (%)** | **Sensitivity (%)** | **Specificity (%)** | **Positive predictive value (%)** | **Negative predictive value (%)** | **Number needed to**  **be screened to**  **identify one case** |
| --- | --- | --- | --- | --- | --- | --- | --- | --- |
| 35.2 | 893 | 31 | 16.70 | 63.27 | 83.73 | 3.47 | 99.60 | 29 |
| 30.6 | 652 | 9 | 28.89 | 81.63 | 71.60 | 2.59 | 99.76 | 39 |
| 27.6 | 713 | 2 | 42.22 | 85.71 | 58.18 | 1.86 | 99.77 | 54 |
| 24.8 | 582 | 4 | 53.10 | 93.88 | 47.27 | 1.62 | 99.88 | 62 |
| 21.9 | 685 | 1 | 65.91 | 95.92 | 34.36 | 1.33 | 99.89 | 75 |
| 19.8 | 335 | 1 | 72.18 | 97.96 | 28.06 | 1.24 | 99.93 | 80 |
| 16.2 | 450 | 1 | 80.59 | 100.00 | 19.59 | 1.14 | 100.00 | 88 |
| 13.6 | 555 | 0 | 90.97 | 100.00 | 9.11 | 1.01 | 100.00 | 99 |
| 9.0 | 339 | 0 | 97.31 | 100.00 | 2.72 | 0.94 | 100.00 | 106 |

^a^ High-risk population is defined as people whose GCRS are higher than the corresponding cutoff.

**Table S14. Risk categories of different gastric lesions in the Yangzhou screening program in sensitivity analysis 1: excluding weak variables in the simplified model ^a^**

| **GCRS**  **cut-offs ^b^** | **Total (n=5,348)** | **GC (n=49) ^c^** | | **DYS (n=163)** | | **IM (n=868)** | | **AG (n=1,626)** | | **Normal (n=2,642)** | |
| --- | --- | --- | --- | --- | --- | --- | --- | --- | --- | --- | --- |
|  | **No. (%)** | **No. (%)** | **CP (%) ^d^** | **No. (%)** | **CP (%)** | **No. (%)** | **No. (%)** | **No. (%)** | **CP (%)** | **No. (%)** | **CP (%)** |
| 9.0~ | 831 (15.54) | 33 (3.97) | 67.35 | 39 (4.69) | 23.93 | 184 (22.14) | 21.20 | 204 (24.55) | 12.55 | 371 (44.65) | 14.04 |
| 7.9-8.9 | 666 (12.45) | 6 (0.90) | 79.59 | 30 (4.50) | 42.33 | 128 (19.22) | 35.94 | 173 (25.98) | 23.19 | 329 (49.40) | 26.50 |
| 7.0-7.8 | 790 (14.77) | 4 (0.51) | 87.76 | 29 (3.67) | 60.12 | 140 (17.72) | 52.07 | 238 (30.13) | 37.82 | 379 (47.97) | 40.84 |
| 6.4-6.9 | 619 (11.57) | 3 (0.48) | 93.88 | 12 (1.94) | 67.48 | 101 (16.32) | 63.71 | 185 (29.89) | 49.20 | 318 (51.37) | 52.88 |
| 5.5-6.3 | 620 (11.59) | 1 (0.16) | 95.92 | 17 (2.74) | 77.91 | 102 (16.45) | 75.46 | 197 (31.77) | 61.32 | 303 (48.87) | 64.35 |
| 5.2-5.4 | 443 (8.28) | 2 (0.45) | 100.00 | 7 (1.58) | 82.21 | 58 (13.09) | 82.14 | 162 (36.57) | 71.28 | 214 (48.31) | 72.45 |
| 4.3-5.1 | 319 (5.96) | 0 (0.00) | 100.00 | 9 (2.82) | 87.73 | 44 (13.79) | 87.21 | 102 (31.97) | 77.55 | 164 (51.41) | 78.65 |
| 3.4-4.2 | 589 (11.01) | 0 (0.00) | 100.00 | 13 (2.21) | 95.71 | 77 (13.07) | 96.08 | 197 (33.45) | 89.67 | 302 (51.27) | 90.08 |
| 2.4-3.3 | 315 (5.89) | 0 (0.00) | 100.00 | 6 (1.90) | 99.39 | 27 (8.57) | 99.19 | 109 (34.60) | 96.37 | 173 (54.92) | 96.63 |
| ~2.3 | 156 (2.92) | 0 (0.00) | 100.00 | 1 (0.64) | 100.00 | 7 (4.49) | 100.00 | 59 (37.82) | 100.00 | 89 (57.05) | 100.00 |

^a^ The simplified model was created based on a subset of strong predictors for gastric cancer (assigned points ≥4.0; including age, sex, education level, intake of salty foods, family history of cancer in first-degree relatives, personal medical history of cancer and peptic ulcer).

^b^ Participants in the Yangzhou screening program were divided into divided into ten groups according to the cut-offs of the GCRS deciles in the CKB cohort. GCRS, gastric cancer risk score.

^c^ GC, gastric cancer. DYS, dysplasia. IM, intestinal metaplasia. AG, atrophic gastritis. “Normal” biopsy report includes the diagnosis of chronic superficial gastritis or no lesions.

^d^ Cumulative proportion (CP) was calculated by dividing the number of each lesion accumulated to this category by the total number of each lesion.

**Table S15.** **Risk categories of different gastric lesions in the Yangzhou screening program in sensitivity analysis 2: integrating lifestyle factors as an index ^a^**

| **GCRS**  **cut-offs ^b^** | **Total (n=5,348)** | **GC (n=49) ^c^** | | **DYS (n=163)** | | **IM (n=868)** | | **AG (n=1,626)** | | **Normal (n=2,642)** | |
| --- | --- | --- | --- | --- | --- | --- | --- | --- | --- | --- | --- |
|  | **No. (%)** | **No. (%)** | **CP (%) ^d^** | **No. (%)** | **CP (%)** | **No. (%)** | **No. (%)** | **No. (%)** | **CP (%)** | **No. (%)** | **CP (%)** |
| 10.7~ | 889 (16.62) | 31 (3.49) | 63.27 | 43 (4.84) | 26.38 | 196 (22.05) | 22.58 | 220 (24.75) | 13.53 | 399 (44.88) | 15.10 |
| 9.4-10.6 | 655 (12.25) | 8 (1.22) | 79.59 | 34 (5.19) | 47.24 | 126 (19.24) | 37.10 | 165 (25.19) | 23.68 | 322 (49.16) | 27.29 |
| 8.4-9.3 | 914 (17.09) | 4 (0.44) | 87.76 | 21 (2.30) | 60.12 | 168 (18.38) | 56.45 | 265 (28.99) | 39.98 | 456 (49.89) | 44.55 |
| 7.8-8.3 | 373 (6.97) | 1 (0.27) | 89.80 | 10 (2.68) | 66.26 | 62 (16.62) | 63.59 | 121 (32.44) | 47.42 | 179 (47.99) | 51.32 |
| 6.8-7.7 | 530 (9.91) | 4 (0.75) | 97.96 | 14 (2.64) | 74.85 | 74 (13.96) | 72.12 | 174 (32.83) | 58.12 | 264 (49.81) | 61.32 |
| 6.0-6.7 | 629 (11.76) | 1 (0.16) | 100.00 | 13 (2.07) | 82.82 | 92 (14.63) | 82.72 | 219 (34.82) | 71.59 | 304 (48.33) | 72.82 |
| 5.3-5.9 | 429 (8.02) | 0 (0.00) | 100.00 | 12 (2.80) | 90.18 | 57 (13.29) | 89.29 | 143 (33.33) | 80.38 | 217 (50.58) | 81.04 |
| 4.3-5.2 | 329 (6.15) | 0 (0.00) | 100.00 | 6 (1.82) | 93.87 | 44 (13.37) | 94.35 | 111 (33.74) | 87.21 | 168 (51.06) | 87.40 |
| 2.9-4.2 | 433 (8.10) | 0 (0.00) | 100.00 | 8 (1.85) | 98.77 | 41 (9.47) | 99.08 | 150 (34.64) | 96.43 | 234 (54.04) | 96.25 |
| ~2.8 | 167 (3.12) | 0 (0.00) | 100.00 | 2 (1.20) | 100.00 | 8 (4.79) | 100.00 | 58 (34.73) | 100.00 | 99 (59.28) | 100.00 |

^a^ The healthy lifestyle index was generated by integrating five modifiable lifestyle factors, i.e., BMI, smoking, alcohol use, consumption of fresh vegetables and fruits, and salty food intake.

^b^ Participants in the Yangzhou screening program were divided into divided into ten groups according to the cut-offs of the GCRS deciles in the CKB cohort. GCRS, gastric cancer risk score.

^c^ GC, gastric cancer. DYS, dysplasia. IM, intestinal metaplasia. AG, atrophic gastritis. “Normal” biopsy report includes the diagnosis of chronic superficial gastritis or no lesions.

^d^ Cumulative proportion (CP) was calculated by dividing the number of each lesion accumulated to this category by the total number of each lesion.

**Table S16. Risk categories of different gastric lesions in the Yangzhou screening program in sensitivity analysis 3: excluding participants who had GC diagnosis within the first year after recruitment**

| **GCRS**  **cut-offs ^a^** | **Total (n=5,348)** | **GC (n=49) ^b^** | | **DYS (n=163)** | | **IM (n=868)** | | **AG (n=1,626)** | | **Normal (n=2,642)** | |
| --- | --- | --- | --- | --- | --- | --- | --- | --- | --- | --- | --- |
|  | **No. (%)** | **No. (%)** | **CP (%) ^c^** | **No. (%)** | **CP (%)** | **No. (%)** | **No. (%)** | **No. (%)** | **CP (%)** | **No. (%)** | **CP (%)** |
| 33.6~ | 916 (17.13) | 31 (3.38) | 63.27 | 42 (4.59) | 25.77 | 211 (23.03) | 24.31 | 222 (24.24) | 13.65 | 410 (44.76) | 15.52 |
| 29.4-33.5 | 691 (12.92) | 9 (1.30) | 81.63 | 36 (5.21) | 47.85 | 121 (17.51) | 38.25 | 181 (26.19) | 24.78 | 344 (49.78) | 28.54 |
| 26.4-29.3 | 661 (12.36) | 2 (0.30) | 85.71 | 20 (3.03) | 60.12 | 120 (18.15) | 52.07 | 210 (31.77) | 37.70 | 309 (46.75) | 40.23 |
| 23.8-26.3 | 619 (11.57) | 4 (0.65) | 93.88 | 14 (2.26) | 68.71 | 107 (17.29) | 64.40 | 183 (29.56) | 48.95 | 311 (50.24) | 52.01 |
| 21.1-23.7 | 605 (11.31) | 1 (0.17) | 95.92 | 12 (1.98) | 76.07 | 96 (15.87) | 75.46 | 193 (31.90) | 60.82 | 303 (50.08) | 63.47 |
| 18.9-21.0 | 414 (7.74) | 0 (0.00) | 95.92 | 10 (2.42) | 82.21 | 49 (11.84) | 81.11 | 147 (35.51) | 69.86 | 208 (50.24) | 71.35 |
| 15.3-18.8 | 449 (8.40) | 2 (0.45) | 100.00 | 11 (2.45) | 88.96 | 64 (14.25) | 88.48 | 155 (34.52) | 79.40 | 217 (48.33) | 79.56 |
| 12.9-15.2 | 525 (9.82) | 0 (0.00) | 100.00 | 11 (2.10) | 95.71 | 66 (12.57) | 96.08 | 169 (32.19) | 89.79 | 279 (53.14) | 90.12 |
| 8.4-12.8 | 325 (6.08) | 0 (0.00) | 100.00 | 6 (1.85) | 99.39 | 27 (8.31) | 99.19 | 114 (35.08) | 96.80 | 178 (54.77) | 96.86 |
| ~8.3 | 143 (2.67) | 0 (0.00) | 100.00 | 1 (0.70) | 100.00 | 7 (4.90) | 100.00 | 52 (36.36) | 100.00 | 83 (58.04) | 100.00 |

^a^ Participants in the Yangzhou screening program were divided into divided into ten groups according to the cut-offs of the GCRS deciles in the CKB cohort. GCRS, gastric cancer risk score.

^b^ GC, gastric cancer. DYS, dysplasia. IM, intestinal metaplasia. AG, atrophic gastritis. “Normal” biopsy report includes the diagnosis of chronic superficial gastritis or no lesions.

^c^ Cumulative proportion (CP) was calculated by dividing the number of each lesion accumulated to this category by the total number of each lesion.

**Table S17. Risk categories of different gastric lesions in the Yangzhou screening program in sensitivity analysis 4: excluding participants who had cancer at baseline**

| **GCRS**  **cut-offs ^a^** | **Total (n=5,312)** | **GC (n=49) ^b^** | | **DYS (n=162)** | | **IM (n=863)** | | **AG (n=1,609)** | | **Normal (n=2,629)** | |
| --- | --- | --- | --- | --- | --- | --- | --- | --- | --- | --- | --- |
|  | **No. (%)** | **No. (%)** | **CP (%) ^c^** | **No. (%)** | **CP (%)** | **No. (%)** | **No. (%)** | **No. (%)** | **CP (%)** | **No. (%)** | **CP (%)** |
| 34.6~ | 878 (16.53) | 31 (3.53) | 63.27 | 41 (4.67) | 25.31 | 201 (22.89) | 23.29 | 212 (24.15) | 13.18 | 393 (44.76) | 14.95 |
| 30.0-34.5 | 648 (12.20) | 9 (1.39) | 81.63 | 35 (5.40) | 46.91 | 114 (17.59) | 36.50 | 164 (25.31) | 23.37 | 326 (50.31) | 27.35 |
| 27.1-29.9 | 715 (13.46) | 2 (0.28) | 85.71 | 22 (3.08) | 60.49 | 137 (19.16) | 52.38 | 221 (30.91) | 37.10 | 333 (46.57) | 40.02 |
| 24.2-27.0 | 668 (12.58) | 4 (0.60) | 93.88 | 14 (2.10) | 69.14 | 110 (16.47) | 65.12 | 199 (29.79) | 49.47 | 341 (51.05) | 52.99 |
| 21.4-24.1 | 578 (10.88) | 1 (0.17) | 95.92 | 13 (2.25) | 77.16 | 91 (15.74) | 75.67 | 184 (31.83) | 60.91 | 289 (50.00) | 63.98 |
| 19.2-21.3 | 372 (7.00) | 2 (0.54) | 100.00 | 7 (1.88) | 81.48 | 45 (12.10) | 80.88 | 132 (35.48) | 69.11 | 186 (50.00) | 71.05 |
| 16.1-19.1 | 414 (7.79) | 0 (0.00) | 100.00 | 11 (2.66) | 88.27 | 58 (14.01) | 87.60 | 146 (35.27) | 78.19 | 199 (48.07) | 78.62 |
| 13.2-16.0 | 572 (10.77) | 0 (0.00) | 100.00 | 12 (2.10) | 95.68 | 73 (12.76) | 96.06 | 186 (32.52) | 89.75 | 301 (52.62) | 90.07 |
| 8.8-13.1 | 323 (6.08) | 0 (0.00) | 100.00 | 6 (1.86) | 99.38 | 27 (8.36) | 99.19 | 111 (34.37) | 96.64 | 179 (55.42) | 96.88 |
| ~8.7 | 144 (2.71) | 0 (0.00) | 100.00 | 1 (0.69) | 100.00 | 7 (4.86) | 100.00 | 54 (37.50) | 100.00 | 82 (56.94) | 100.00 |

^a^ Participants in the Yangzhou screening program were divided into divided into ten groups according to the cut-offs of the GCRS deciles in the CKB cohort. GCRS, gastric cancer risk score.

^b^ GC, gastric cancer. DYS, dysplasia. IM, intestinal metaplasia. AG, atrophic gastritis. “Normal” biopsy report includes the diagnosis of chronic superficial gastritis or no lesions.

^c^ Cumulative proportion (CP) was calculated by dividing the number of each lesion accumulated to this category by the total number of each lesion.

**Table S18. Risk categories of different gastric lesions in the Yangzhou screening program in sensitivity analysis 5: competing risk model**

| **GCRS**  **cut-offs ^a^** | **Total (n=5,348)** | **GC (n=49) ^b^** | | **DYS (n=163)** | | **IM (n=868)** | | **AG (n=1,626)** | | **Normal (n=2,642)** | |
| --- | --- | --- | --- | --- | --- | --- | --- | --- | --- | --- | --- |
|  | **No. (%)** | **No. (%)** | **CP (%) ^c^** | **No. (%)** | **CP (%)** | **No. (%)** | **No. (%)** | **No. (%)** | **CP (%)** | **No. (%)** | **CP (%)** |
| 35.8~ | 899 (16.81) | 31 (3.45) | 63.27 | 42 (4.67) | 25.77 | 206 (22.91) | 23.73 | 223 (24.81) | 13.71 | 397 (44.16) | 15.03 |
| 31.2-35.7 | 646 (12.08) | 9 (1.39) | 81.63 | 33 (5.11) | 46.01 | 113 (17.49) | 36.75 | 165 (25.54) | 23.86 | 326 (50.46) | 27.37 |
| 28.1-31.1 | 711 (13.29) | 2 (0.28) | 85.71 | 23 (3.23) | 60.12 | 131 (18.42) | 51.84 | 222 (31.22) | 37.52 | 333 (46.84) | 39.97 |
| 25.3-28.0 | 596 (11.14) | 4 (0.67) | 93.88 | 12 (2.01) | 67.48 | 94 (15.77) | 62.67 | 174 (29.19) | 48.22 | 312 (52.35) | 51.78 |
| 22.4-25.2 | 667 (12.47) | 1 (0.15) | 95.92 | 15 (2.25) | 76.69 | 114 (17.09) | 75.81 | 212 (31.78) | 61.25 | 325 (48.73) | 64.08 |
| 20.2-22.3 | 344 (6.43) | 1 (0.29) | 97.96 | 6 (1.74) | 80.37 | 41 (11.92) | 80.53 | 123 (35.76) | 68.82 | 173 (50.29) | 70.63 |
| 16.6-20.1 | 447 (8.36) | 1 (0.22) | 100.00 | 13 (2.91) | 88.34 | 62 (13.87) | 87.67 | 157 (35.12) | 78.47 | 214 (47.87) | 78.73 |
| 13.9-16.5 | 557 (10.42) | 0 (0.00) | 100.00 | 12 (2.15) | 95.71 | 72 (12.93) | 95.97 | 179 (32.14) | 89.48 | 294 (52.78) | 89.86 |
| 9.2-13.8 | 337 (6.30) | 0 (0.00) | 100.00 | 6 (1.78) | 99.39 | 28 (8.31) | 99.19 | 117 (34.72) | 96.68 | 186 (55.19) | 96.90 |
| ~9.1 | 144 (2.69) | 0 (0.00) | 100.00 | 1 (0.69) | 100.00 | 7 (4.86) | 100.00 | 54 (37.50) | 100.00 | 82 (56.94) | 100.00 |

^a^ Participants in the Yangzhou screening program were divided into divided into ten groups according to the cut-offs of the GCRS deciles in the CKB cohort. GCRS, gastric cancer risk score.

^b^ GC, gastric cancer. DYS, dysplasia. IM, intestinal metaplasia. AG, atrophic gastritis. “Normal” biopsy report includes the diagnosis of chronic superficial gastritis or no lesions.

^c^ Cumulative proportion (CP) was calculated by dividing the number of each lesion accumulated to this category by the total number of each lesion.

**Table S19. The GCRS and corresponding 3-year, 5-year, and 10-year risk of incident GC derived from the CKB cohort**

| **GCRS ^a^** | **3-year risk, %** | **5-year risk, %** | **10-year risk, %** |  | **GCRS** | **3-year risk, %** | **5-year risk, %** | **10-year risk, %** |
| --- | --- | --- | --- | --- | --- | --- | --- | --- |
| 0 | 0.01 | 0.02 | 0.05 |  | 34 | 0.44 | 0.73 | 1.61 |
| 1 | 0.02 | 0.03 | 0.06 |  | 35 | 0.49 | 0.80 | 1.78 |
| 2 | 0.02 | 0.03 | 0.06 |  | 36 | 0.54 | 0.89 | 1.97 |
| 3 | 0.02 | 0.03 | 0.07 |  | 37 | 0.60 | 0.98 | 2.18 |
| 4 | 0.02 | 0.04 | 0.08 |  | 38 | 0.67 | 1.09 | 2.42 |
| 5 | 0.02 | 0.04 | 0.09 |  | 39 | 0.74 | 1.20 | 2.67 |
| 6 | 0.03 | 0.04 | 0.10 |  | 40 | 0.82 | 1.33 | 2.96 |
| 7 | 0.03 | 0.05 | 0.11 |  | 41 | 0.90 | 1.47 | 3.27 |
| 8 | 0.03 | 0.05 | 0.12 |  | 42 | 1.00 | 1.63 | 3.62 |
| 9 | 0.04 | 0.06 | 0.13 |  | 43 | 1.10 | 1.80 | 4.00 |
| 10 | 0.04 | 0.06 | 0.14 |  | 44 | 1.22 | 2.00 | 4.43 |
| 11 | 0.04 | 0.07 | 0.16 |  | 45 | 1.35 | 2.21 | 4.90 |
| 12 | 0.05 | 0.08 | 0.17 |  | 46 | 1.49 | 2.44 | 5.42 |
| 13 | 0.05 | 0.09 | 0.19 |  | 47 | 1.65 | 2.70 | 6.00 |
| 14 | 0.06 | 0.10 | 0.21 |  | 48 | 1.83 | 2.99 | 6.63 |
| 15 | 0.07 | 0.11 | 0.24 |  | 49 | 2.02 | 3.31 | 7.34 |
| 16 | 0.07 | 0.12 | 0.26 |  | 50 | 2.24 | 3.66 | 8.12 |
| 17 | 0.08 | 0.13 | 0.29 |  | 51 | 2.48 | 4.05 | 8.98 |
| 18 | 0.09 | 0.14 | 0.32 |  | 52 | 2.74 | 4.48 | 9.93 |
| 19 | 0.10 | 0.16 | 0.35 |  | 53 | 3.03 | 4.95 | 10.99 |
| 20 | 0.11 | 0.18 | 0.39 |  | 54 | 3.35 | 5.48 | 12.16 |
| 21 | 0.12 | 0.2 | 0.43 |  | 55 | 3.71 | 6.06 | 13.45 |
| 22 | 0.13 | 0.22 | 0.48 |  | 56 | 4.10 | 6.70 | 14.88 |
| 23 | 0.15 | 0.24 | 0.53 |  | 57 | 4.54 | 7.42 | 16.46 |
| 24 | 0.16 | 0.26 | 0.59 |  | 58 | 5.02 | 8.21 | 18.21 |
| 25 | 0.18 | 0.29 | 0.65 |  | 59 | 5.56 | 9.08 | 20.15 |
| 26 | 0.20 | 0.32 | 0.72 |  | 60 | 6.15 | 10.04 | 22.29 |
| 27 | 0.22 | 0.36 | 0.80 |  | 61 | 6.80 | 11.11 | 24.65 |
| 28 | 0.24 | 0.4 | 0.88 |  | 62 | 7.52 | 12.29 | 27.27 |
| 29 | 0.27 | 0.44 | 0.97 |  | 63 | 8.32 | 13.60 | 30.17 |
| 30 | 0.30 | 0.49 | 1.08 |  | 64 | 9.21 | 15.04 | 33.38 |
| 31 | 0.33 | 0.54 | 1.19 |  | 65 | 10.19 | 16.64 | 36.93 |
| 32 | 0.36 | 0.59 | 1.32 |  | 66 | 11.27 | 18.41 | 40.85 |
| 33 | 0.40 | 0.66 | 1.46 |  | 67 | 12.47 | 20.36 | 45.19 |

^a^ The GCRSs were rounded to integers.

**Fig. S1. Study design and eligible participants’ selection procedures in three studies**


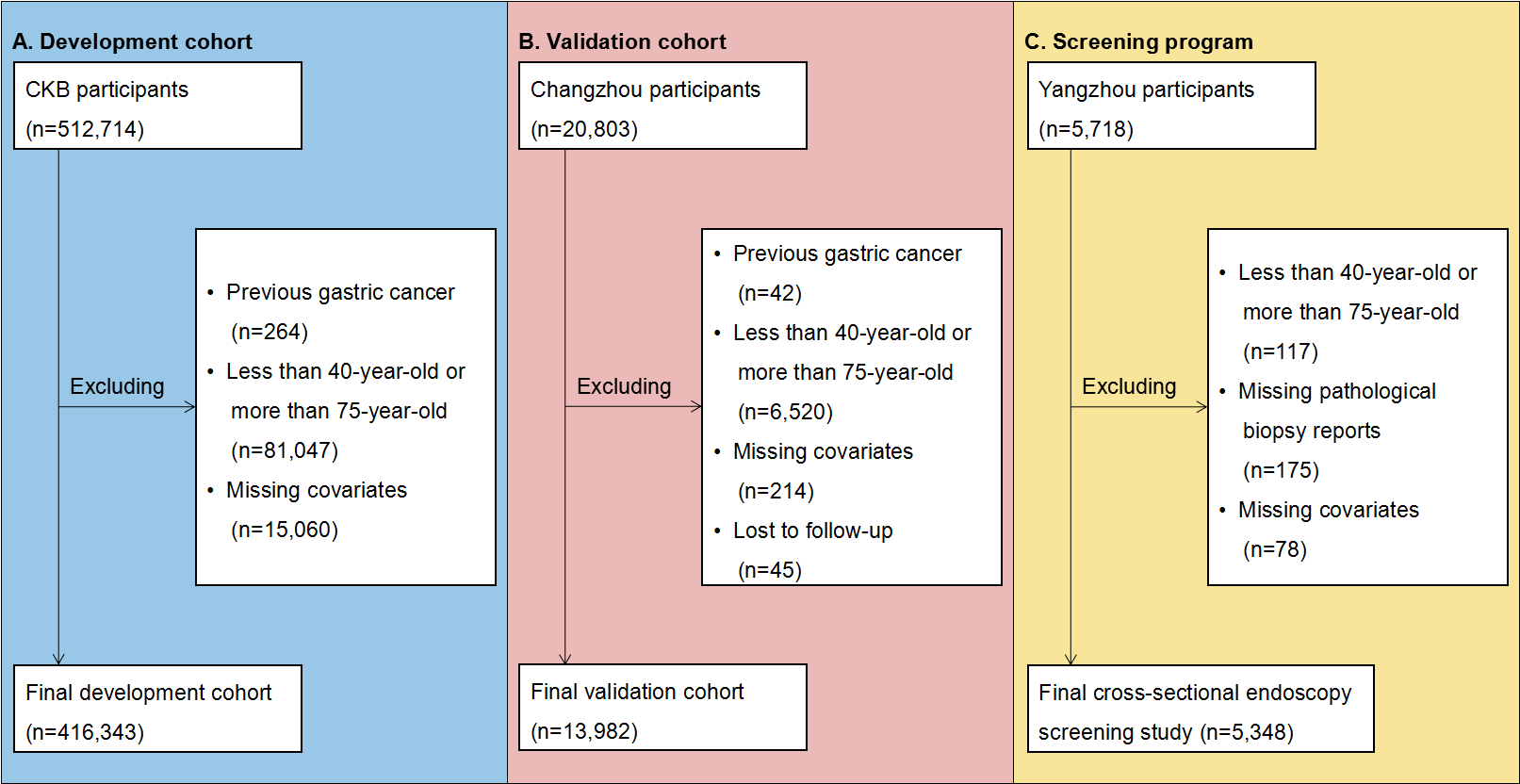


**Fig. S2. The relationship of the GCRS with incident GC risk in the CKB cohort.**


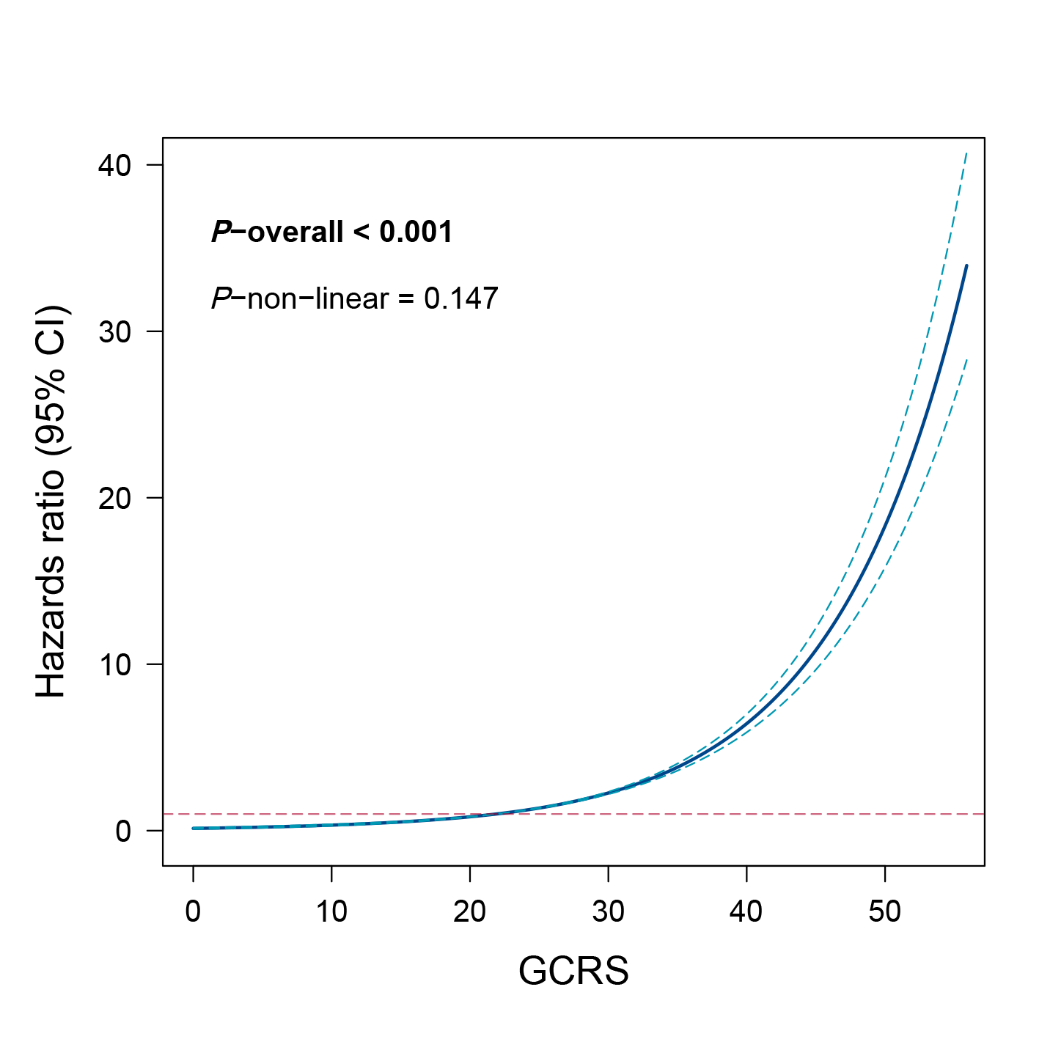


Linear relationship between the GCRS and gastric cancer risk was assessed by using a restricted cubic spline analysis, and hazards ratios and 95% CIs were estimated. GCRS, gastric cancer risk score.

**Fig. S3. The relationship of the GCRS with incident GC risk in the Changzhou cohort.**


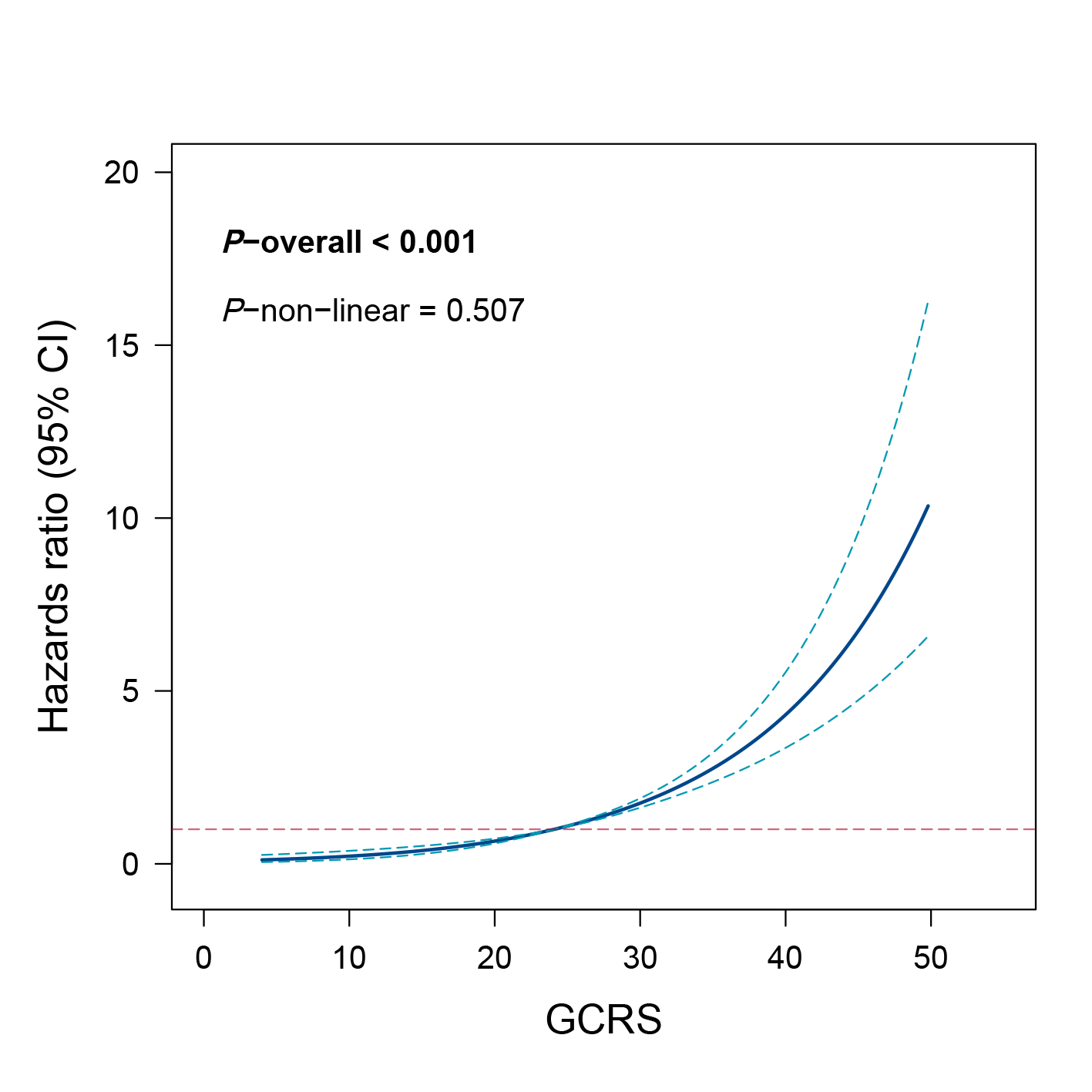


Linear relationship between the GCRS and gastric cancer risk was assessed by using a restricted cubic spline analysis, and hazards ratios and 95% CIs were estimated. GCRS, gastric cancer risk score.

**Fig. S4. Calibration and discrimination of the GCRS in sensitivity analysis 1: excluding weak variables in the simplified model**


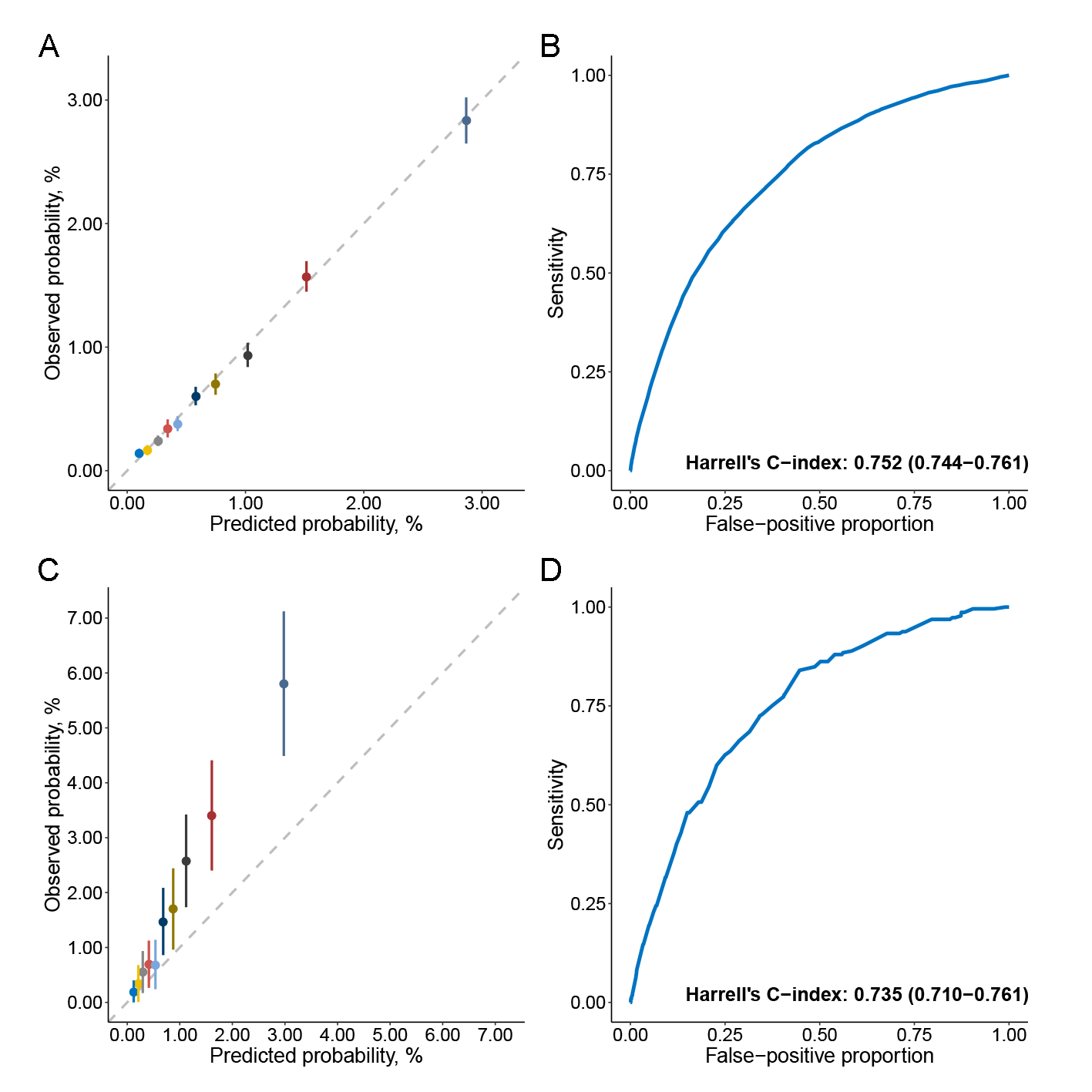


The simplified model was created based on a subset of strong predictors for gastric cancer (assigned points ≥4.0; including age, sex, education level, intake of salty foods, family history of cancer in first-degree relatives, personal medical history of cancer and peptic ulcer). The observed 10-year probability of GC with 95% CIs was estimated by the Kaplan-Meier method within deciles of GCRS-based model predicted probability in the CKB cohort (A) and Changzhou cohort (C). The *R^2^* coefficient was 0.997 for the CKB cohort and 0.988 for the Changzhou cohort. Receiver operating characteristic curve at 10 years in the CKB cohort (B) and Changzhou cohort (D). The Harrell’s C-index was 0.752 [0.744-0.761] for the CKB cohort and 0.735 [0.710-0.761] for the Changzhou cohort. GCRS, gastric cancer risk score. CKB, China Kadoorie Biobank.

**Fig. S5. Calibration and discrimination of the GCRS in sensitivity analysis 2: integrating lifestyle factors as an index**


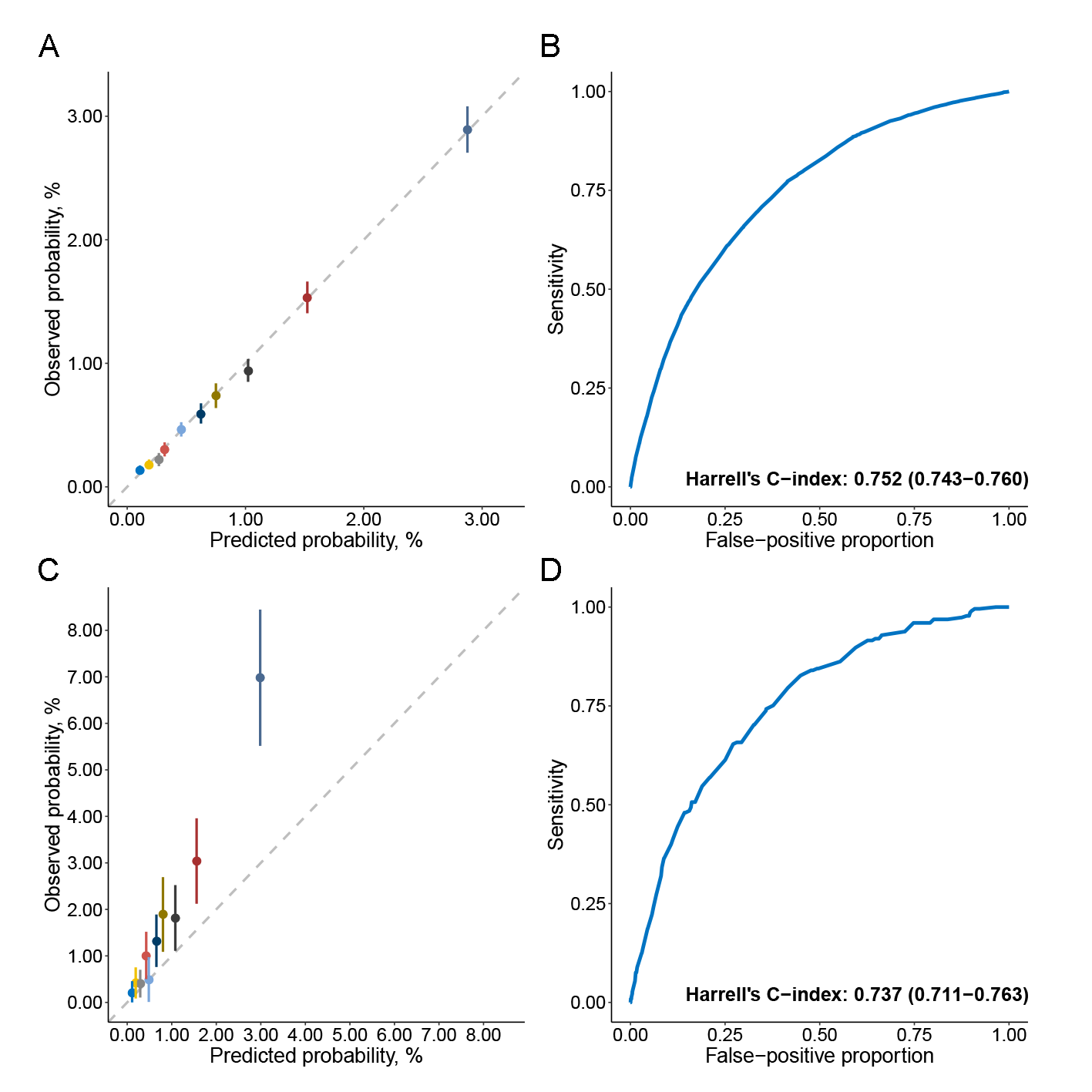


The healthy lifestyle index was generated by integrating five modifiable lifestyle factors, i.e., BMI, smoking, alcohol use, consumption of fresh vegetables and fruits, and salty food intake. The observed 10-year probability of GC with 95% CIs was estimated by the Kaplan-Meier method within deciles of GCRS-based model predicted probability in the CKB cohort (A) and Changzhou cohort (C). The *R^2^* coefficient was 0.999 for the CKB cohort and 0.979 for the Changzhou cohort. Receiver operating characteristic curve at 10 years in the CKB cohort (B) and Changzhou cohort (D). The Harrell’s C-index was 0.752 [0.743-0.760] for the CKB cohort and 0.737 [0.711-0.763] for the Changzhou cohort. GCRS, gastric cancer risk score. CKB, China Kadoorie Biobank.

**Fig. S6. Calibration and discrimination of the GCRS in sensitivity analysis 3: excluding participants who had GC diagnosis within the first year after recruitment**

**
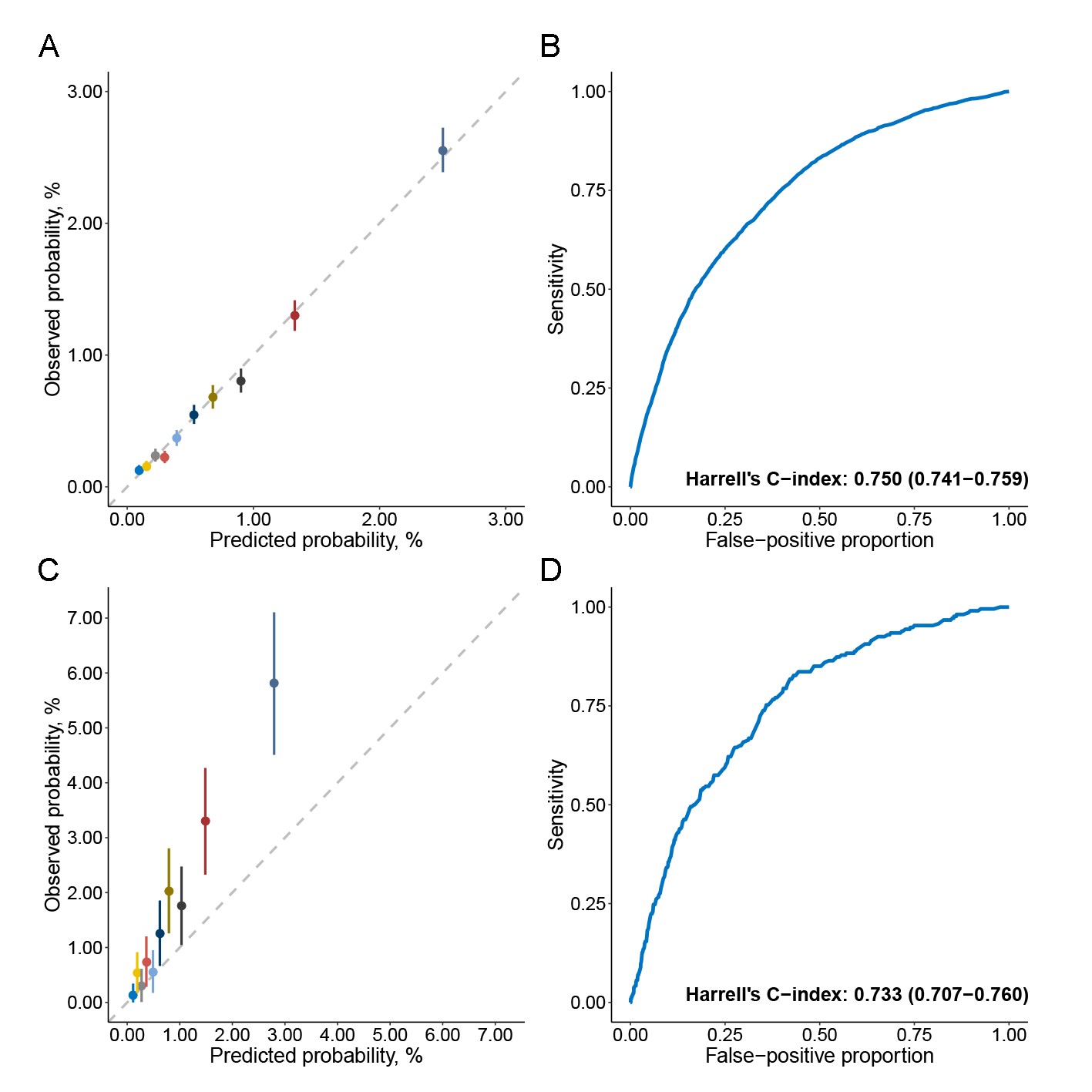
**

The observed 10-year probability of GC with 95% CIs was estimated by the Kaplan-Meier method within deciles of GCRS-based model predicted probability in the CKB cohort (A) and Changzhou cohort (C) after excluding participants who had GC diagnosis within the first year after recruitment. The *R^2^* coefficient was 0.996 for the CKB cohort and 0.978 for the Changzhou cohort. Receiver operating characteristic curve at 10 years in the CKB cohort (B) and Changzhou cohort (D). The Harrell’s C-index was 0.750 [0.741-0.759] for the CKB cohort and 0.733 [0.707-0.760] for the Changzhou cohort. GCRS, gastric cancer risk score. CKB, China Kadoorie Biobank.

**Fig. S7. Calibration and discrimination of the GCRS in sensitivity analysis 4: excluding participants who had cancer at baseline**

**
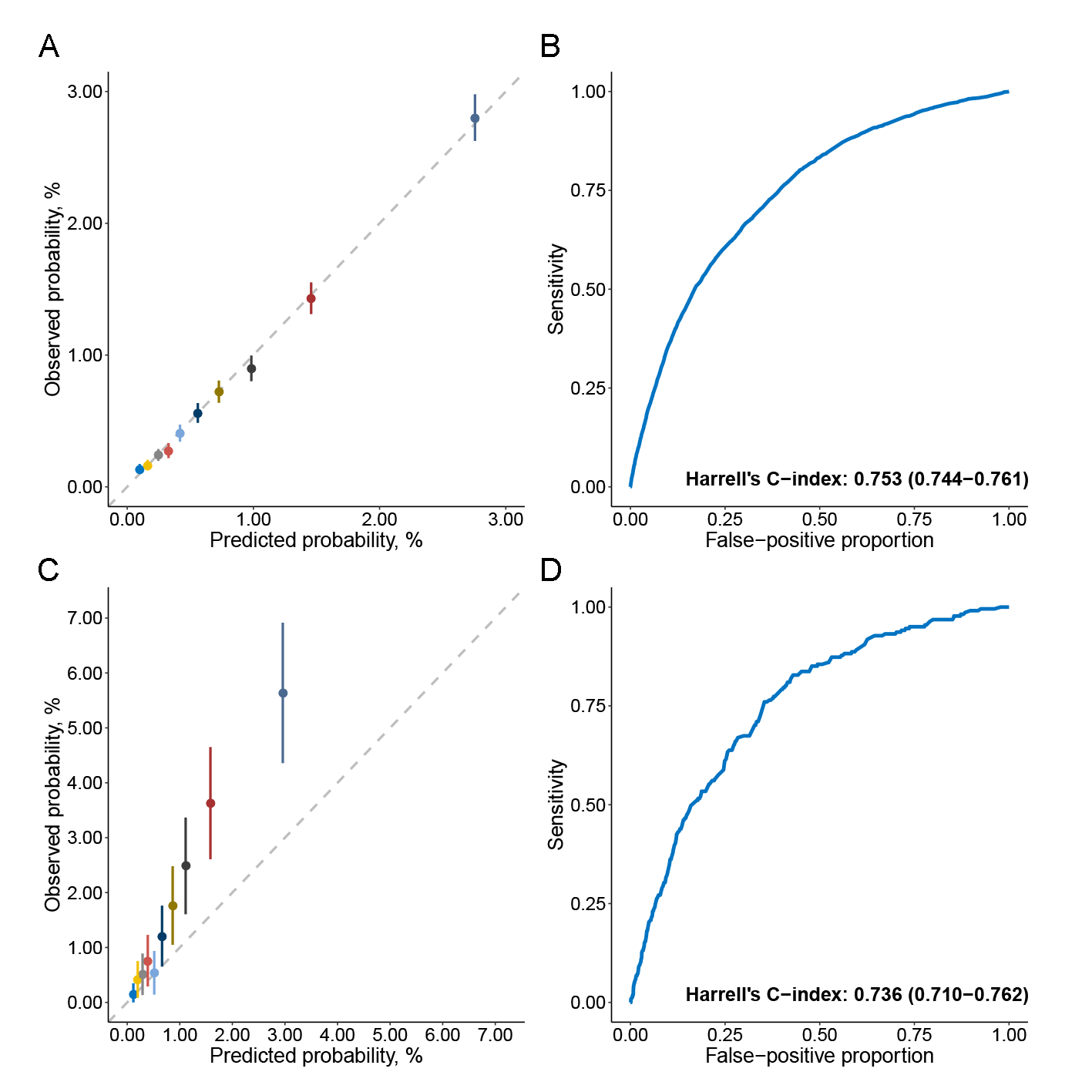
**

The observed 10-year probability of GC with 95% CIs was estimated by the Kaplan-Meier method within deciles of GCRS-based model predicted probability in the CKB cohort (A) and Changzhou cohort (C) after excluding participants who had cancer at baseline. The *R^2^* coefficient was 0.998 for the CKB cohort and 0.978 for the Changzhou cohort. Receiver operating characteristic curve at 10 years in the CKB cohort (B) and Changzhou cohort (D). The Harrell’s C-index was 0.753 [0.744-0.761] for the CKB cohort and 0.736 [0.710-0.762] for the Changzhou cohort. GCRS, gastric cancer risk score. CKB, China Kadoorie Biobank.

**Fig. S8. Calibration and discrimination of the GCRS in sensitivity analysis 5: competing risk model**

**
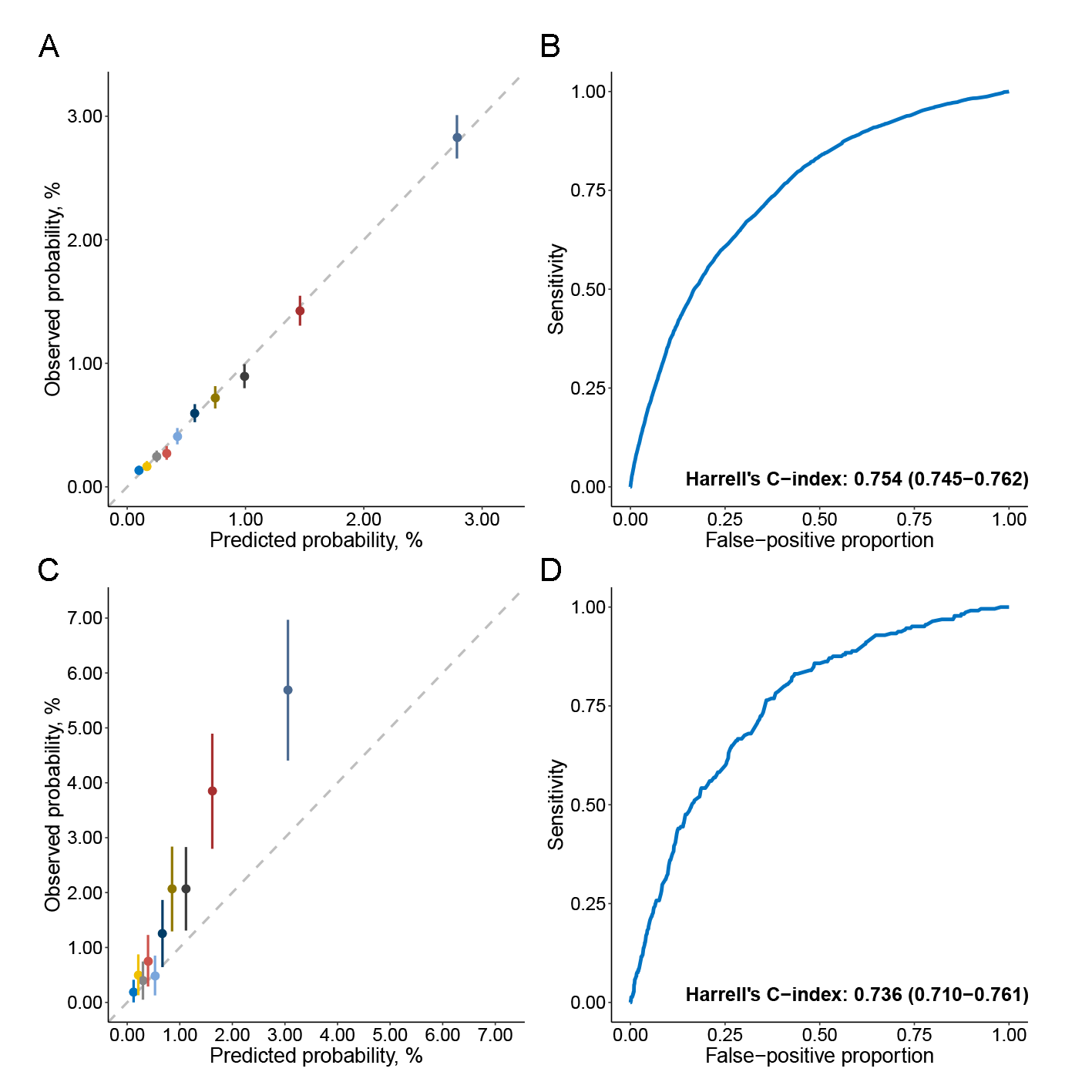
**

The observed 10-year probability of GC with 95% CIs was estimated by the Kaplan-Meier method within deciles of GCRS-based model predicted probability in the CKB cohort (A) and Changzhou cohort (C) in the competing risk model. The *R^2^* coefficient was 0.997 for the CKB cohort and 0.963 for the Changzhou cohort. Receiver operating characteristic curve at 10 years in the CKB cohort (B) and Changzhou cohort (D). The Harrell’s C-index was 0.754 [0.745-0.762] for the CKB cohort and 0.736 [0.710-0.761] for the Changzhou cohort. GCRS, gastric cancer risk score. CKB, China Kadoorie Biobank.
